# Ancient DNA reveals a two-clanned matrilineal community in Neolithic China

**DOI:** 10.1038/s41586-025-09103-x

**Published:** 2025-06-04

**Authors:** Jincheng Wang, Shi Yan, Zhenguang Li, Jinguo Zan, Yichao Zhao, Jin Zhao, Kui Chen, Xueye Wang, Ting Ji, Cheng Zhang, Tingyu Yang, Tianming Zhang, Rui Qiao, Meilin Guo, Zongyue Rao, Jiashuo Zhang, Guanbo Wang, Zhiyu Ran, Chen Duan, Fan Zhang, Yin Song, Xiaohong Wu, Ruth Mace, Bo Sun, Yuhong Pang, Yanyi Huang, Hai Zhang, Chao Ning

**Affiliations:** 1 School of Archaeology and Museology, Peking University, Beijing, China; 2 Biomedical Pioneering Innovation Center, Peking University, Beijing, China; 3 Peking-Tsinghua Center for Life Sciences, Peking University, Beijing, China; 4 College of Chemistry and Molecular Engineering, Beijing National Laboratory for Molecular Sciences, Peking University, Beijing, China; 5 School of Ethnology and Sociology, Minzu University of China, Beijing, China; 6 Shandong Institute of Cultural Relics and Archaeology, Jinan, China; 7 Dongying Museum, Dongying, China; 8 Cultural Relics Protection and Archaeological Research Institute of Shandong, Zibo, China; 9 Center for Archaeological Science, Sichuan University, Chengdu, China; 10 School of Archaeology and Museology, Sichuan University, Chengdu, China; 11 Key Laboratory of Animal Ecology and Conservation Biology, Institute of Zoology, Chinese Academy of Sciences, Beijing, China; 12 School of Archaeology, Jilin University, Changchun, China; 13 Institute of Chemical Biology, Shenzhen Bay Laboratory, Shenzhen, China; 14 Key Laboratory of Archaeological Science (Peking University), Ministry of Education, Beijing, China; 15 Department of Anthropology, University College London, London, UK; 16 Institute for Advanced Study in Toulouse, Université de Toulouse 1 Capitole, Toulouse, France; 17 Center for the Studies of Chinese Archaeology, Peking University, Beijing, China

**Keywords:** Population genetics, Social evolution, Anthropology, Archaeology

## Abstract

Studies of ancient DNA from cemeteries provide valuable insights into early human societies, and have strongly indicated patrilocality^1–10^. Here, we analysed ancient DNA alongside archaeological contexts and multiple stable isotopic data from 60 individuals in 2 separate cemeteries at the Fujia archaeological site in eastern China, dating between 2750 and 2500 bce. Our findings suggest the existence of an early-described matrilineal community in the Neolithic period, characterized by high endogamy and a population practicing millet agriculture near the coast. Evidence of intermarriage between individuals in the two cemeteries and the presence of both primary and secondary burials, organized strictly according to maternal clans, underscore a strong sense of social cohesion and identity at Fujia. Bayesian modelling of radiocarbon dates indicates that the two cemeteries were used for approximately 250 years, implying a stable matrilineal lineage spanning at least 10 generations. This study contributes to the ongoing debate in anthropology and archaeology^11^, not only suggesting the existence of a matrilineal society in early human history but also revealing a pair of Neolithic cemeteries organized around two matrilineal clans, furthering our understanding of the early evolution of human societies through kinship systems.

## Abstract (web summary)

Analysis of ancient DNA, stable isotope data and archaeological evidence from the Fujia archaeological site in eastern China suggests it was populated by a matrilineal Neolithic community between 2750 and 2500 bc.

## Main

**Fig. 1:**
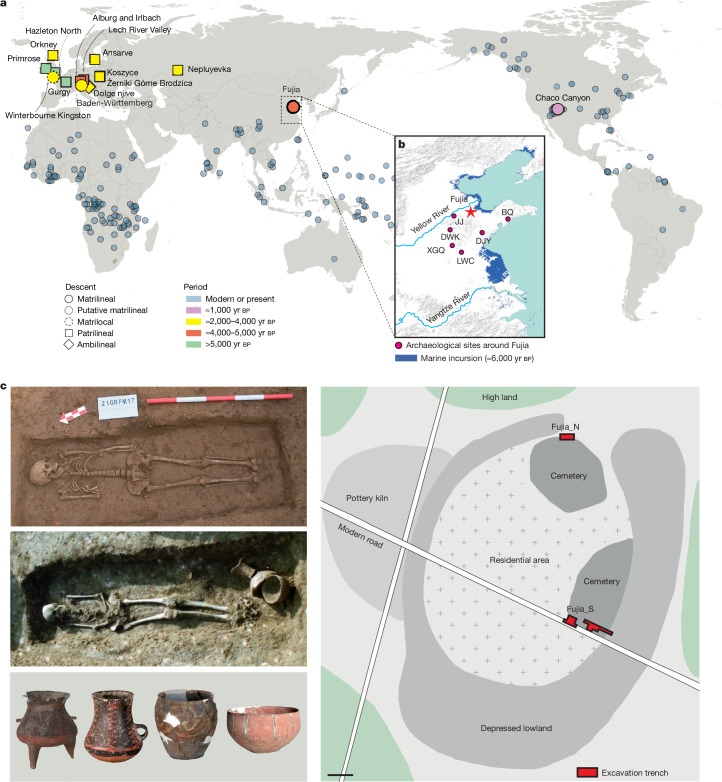
Fujia archaeological site and ancient and present-day populations associated with Fujia. **a**, Spatial distribution of kinship organization in global populations, adapted from D-PLACE^61,62^ and in the literature. Kinship structures in ancient populations are represented using different colours and symbols, which differentiate various types such as matrilineal and patrilineal systems across different regions and time periods. The world map was created using the R package maps (v3.4.2). **b**, Geographic location of the Fujia archaeological site in relation to the marine incursion around 6,000 years before present. Surrounding archaeological sites from the same period as Fujia, including Jiaojia (JJ), Dawenkou (DWK), Dongjiaying (DJY), Liangwangcheng (LWC), Xigongqiao (XGQ) and Beiqian (BQ), are marked to provide context. The base map was obtained from the Word Terrain Base domain map dataset (https://www.arcgis.com/home/item.html?id=c61ad8ab017d49e1a82f580ee1298931) and created with ArcGIS v10.2. Sources: Esri, USGS, NOAA. **c**, Painted pottery (bottom left) from the Fujia cemetery, as well as from the southern grave (FJ_S21; middle left) and northern grave (FJ_N07; top left). The right panel illustrates the layout and features of the Fujia archaeological site, highlighting residential areas, pottery kilns and the two cemeteries. Scale bar, 50 m.

Whether early human societies were organized by patrilineal or matrilineal descent is a key but debated question. Over the past two decades, many efforts to address this question have been made by archaeologists, linguistics and geneticists, but no common consensus has been reached^12–18^. Phylogenetic studies of cultural evolution do not support rigid, unidirectional hypotheses about human social evolution, but show more dynamic and flexible kinship systems changing repeatedly, often responding to environmental and economic changes, with the spread of pastoralism and agriculture often favouring patrilocality^13,19,20^. Although matrilineal social organization is now rare, especially in China, it is possible that it was more common in the past. Recent advances in genetic analyses and Bayesian modelling of language families’ common ancestors have yielded new insights into human social structures^13–18^. However, almost all genome-wide studies up to now examining the Neolithic and Bronze Age periods have consistently supported patrilocality and patrilineality^2–10^, and the existence and locations of matriliny in the remote or prehistoric past are still largely unknown. So far, the only ancient matrilineal society confirmed by genomic analysis is the elite Chaco Canyon dynasty in North America, dating from 800 to 1,300 ce (ref. ^21^). There is also suggestive evidence of possible matrilineal descent among some Celtic elites from 616 to 200 bce in southern Germany^22^ as well as matrilocality in Iron Age Britain, where Durotriges burials reveal dominant maternal lineages and male in-migration^23^ (Fig. 1a).

In this study, we obtained genome-wide data using optimized ancient DNA methods to reconstruct the precise genetic relationships of ancient individuals buried at the Fujia archaeological site in eastern China, dating between 2750 and 2500 bce. Our results presented comprehensive genomic evidence of a two-clanned matrilineal community at the Fujia site, beyond data from just sequencing the hypervariable region of mitochondrial DNA (mtDNA)^24^. We also implemented an interdisciplinary approach involving stable isotopes, osteological classification, palaeoenvironment analyses and Bayesian modelling of radiocarbon dates to provide insights into their subsistence practices, local environment, social complexity and cemetery usage duration. The Fujia case not only confirms the existence of a Neolithic matrilineal society, filling a gap in the evidence of ancient matriliny, but also provides its environmental–societal context, expanding our understanding of the diversity and complexity of the matrilineal kinship organization of early human societies.

## Background and sampling at Fujia site

Fujia (37° 2′ 20.19″ N, 118° 23′ 39.63″ E) is a Neolithic archaeological site located in Shandong province in eastern China, north of the Tai-Yi Mountains and near the southern coast of the Bohai Sea (Fig. 1b). Through archaeological surveys and excavations, Fujia has been identified as a late Dawenkou culture site (Fig. 1c and Supplementary Note 1). It covers approximately 37 hectares^25–27^ (Supplementary Table 1 and Supplementary Fig. 1), and was excavated three times, in 1985, 1995 and 2021 (ref. ^26^). Positioned on an elevated ground surrounded by lowlands near the sea, Fujia features centrally concentrated residential areas, with pottery kilns primarily situated on a small, elevated area to the west. Two separate cemeteries, labelled Fujia_N and Fujia_S, were unearthed in the north and southeast of the site accordingly (Fig. 1c). More than 500 burials have been excavated at the site, with available radiocarbon dates between 2750 and 2500 cal bc (Supplementary Table 2 and Supplementary Fig. 3). Bayesian modelling of accelerator mass spectrometry dates from the cemeteries has revealed that the burial practice spanned about 250 years and encompassed at least 10 generations (Supplementary Tables 3 and 4, Supplementary Figs. 4 and 5 and Supplementary Note 2). Osteological classifications provide insights into burial practices at Fujia. Of the 295 identified individuals in Fujia_S, 58% are male and 42% female. Among all of the burials in Fujia_S, 35% of the 355 individuals were identified as secondary burials, with joint burials involving multiple skeletons accounting for 12%. On the basis of age estimations of the skeletons excavated in 1995, the even distribution of all age ranges (mean age = 23.4 years) indicates no age bias in Fujia’s burial practices (Supplementary Note 1). Notably, a male individual aged 35–45 at Fujia_S exhibited evidence of craniotomy on his skull^28,29^.

For this study, 66 individuals with intact skulls were selected (15 from Fujia_N and 51 from Fujia_S; Supplementary Data 1). After ancient DNA authentication, six individuals were removed from the dataset owing to either a low level of DNA coverage or a high level of modern human DNA contamination, resulting in valid data for 14 individuals from Fujia_N and 46 individuals from Fujia_S. Population genetic analyses showed genetic homogeneity among these individuals (Supplementary Figs. 6–8 and Supplementary Note 3). We analysed the strontium isotope ratios (^87^Sr/^86^Sr) of 32 human tooth enamel samples and compared them with newly obtained isotopic data from archaeological animal remains and modern wild plants collected across the study area. Additionally, we obtained 29 authenticated carbon (*δ*^13^C, with *δ*^13^C = [(^13^C/^12^C)_sample_/(^13^C/^12^C)_standard_] − 1) and nitrogen (*δ*^15^N) isotope values from human bone and dentine collagen. New radiocarbon dates were reported from 19 individuals (Supplementary Data 2).

## Matrilineal clan community at Fujia

**Fig. 2:**
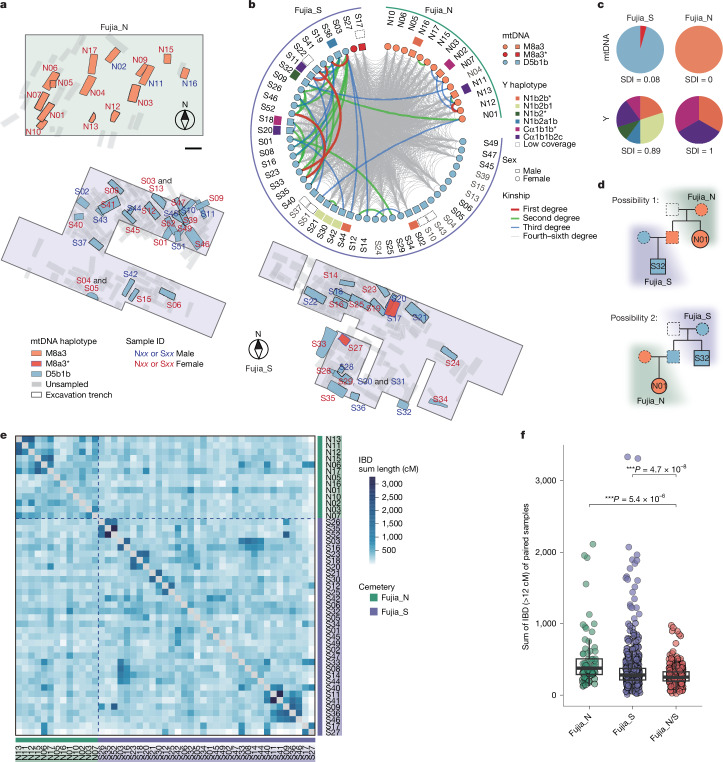
Uniparental genetic marker and biological relatedness of the Fujia individuals. **a**, A zoom-in view of the burials excavated from the Fujia site, showing the spatial distribution of the sampled individuals, colour-coded according to their mtDNA haplotypes and annotated with labels indicating the genetic sex of each individual. Scale bar, 2 m. **b**, Genetic relatedness of the Fujia individuals. The innermost circle represents the genetic sex of each individual: squares for male and circles for female, along with mitochondrial haplotypes indicated by colour. The middle layer indicates Y-chromosome haplotypes for male individuals. The outermost layer shows each individual ID. Lines within the inner circle illustrate genetic kinship among individuals, estimating first- to third-degree relationships from KIN, and fourth- to sixth-degree relationships from ancIBD. Sample names in grey represent individuals excluded from the IBD analysis. **c**, Pie charts showing the compositions and frequencies of mtDNA and Y-chromosome haplotypes, highlighting low mtDNA diversity and high Y-chromosome diversity. **d**, Examples of burials adhering to maternal lineages involving a pair of second-degree relatives, N01 and S32, who have different mtDNA. In both cases, their burials correspond to their respective maternal lineages, as inferred from the pedigrees. **e**, Heat map showing pairwise IBD sharing between individuals with more than 240,000 single nucleotide polymorphisms (*n* = 44), illustrating extensive kinship between Fujia_N and Fujia_S. **f**, The pairwise IBD sharing of paired individuals from inter-cemetery groups (Fujia_N, *n* = 78 pairs; Fujia_S, *n* = 561 pairs) and intra-cemetery groups (Fujia_N/S, *n* = 442 pairs), revealing closer biological relatedness within the same cemetery. Box plots show the median (centre line), first and third quartiles (lower and upper hinges), and whiskers extending to 1.5× interquartile range from the hinges. ***P value < 0.001, from two-sided *t*-test.

**Extended Data Fig. 1:**
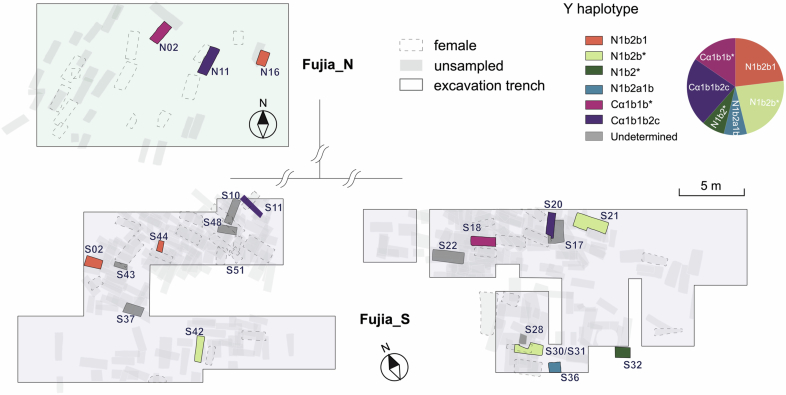
Spatial distribution of the Y-chromosome haplotypes in Fujia males. Spatial distribution of Y-chromosome haplotypes among Fujia males, represented by distinct colors in pie charts that depict the composition and frequency of each haplotype. Specific haplotypes such as N1b2b* (not N1b2b1), N1b2* (not N1b2b), and Cα1b1b* (not Cα1b1b2c), as detailed in Supplementary Data S1, are included. This visualization reveals the presence of identical Y haplotypes across both cemeteries, marking a notable contrast to the mtDNA patterns, which indicate more distinct lineage separations between cemeteries.

The examination of maternally inherited mtDNA and paternally inherited Y chromosomes revealed a clear correspondence between cemetery locations and mtDNA haplotypes, independent of the individual’s genetic sex (Fig. 2a). All 14 individuals (3 male individuals; 11 female individuals) excavated from Fujia_N were found to possess mtDNA haplogroup M8a3, with a Simpson’s diversity index (SDI) value of 0. By contrast, a distinct mtDNA haplogroup, D5b1b, was identified as prevalent in Fujia_S, possessed by 44 (15 male individuals; 29 female individuals) of 46 individuals (95.65%), resulting in an SDI value of 0.08 (Fig. 2b,c). Furthermore, individuals interred in the same cemetery who shared identical mitochondrial haplotypes also exhibit identical mtDNA sequences, suggesting a shared maternal lineage (Supplementary Data 3). Although the remaining two individuals from Fujia_S, S17 and S27, belonged to the mitochondrial haplogroup M8a3, their mtDNA sequences differed by six base pairs from those of Fujia_N individuals, indicating a distinct maternal lineage (Supplementary Data 3). In contrast to the observed mtDNA patterns, results from analysis of the Y chromosome revealed a notably high degree of haplotype diversity (Fig. 2b,c, Extended Data Fig. 1 and Supplementary Data 1). Specifically, a detailed examination of 13 male samples (3 from Fujia_N; 10 from Fujia_S) with sufficient coverage (>0.2×) on the Y chromosome enabled the determination of Y-chromosome haplogroups. This analysis revealed that the 10 male individuals from Fujia_S showed a markedly high lineage diversity, with 6 unique Y lineages identified (SDI = 0.89; Fig. 2b,c and Supplementary Data 1), and the 3 male individuals from Fujia_N exhibited 3 distinct Y haplogroups (SDI = 1), with the 3 sequences being indistinguishable from those identified in Fujia_S. By integrating mtDNA and Y-chromosome analyses, we provide evidence that most individuals at Fujia, irrespective of their sex, were buried according to their maternal lineages.

**Extended Data Fig. 2:**
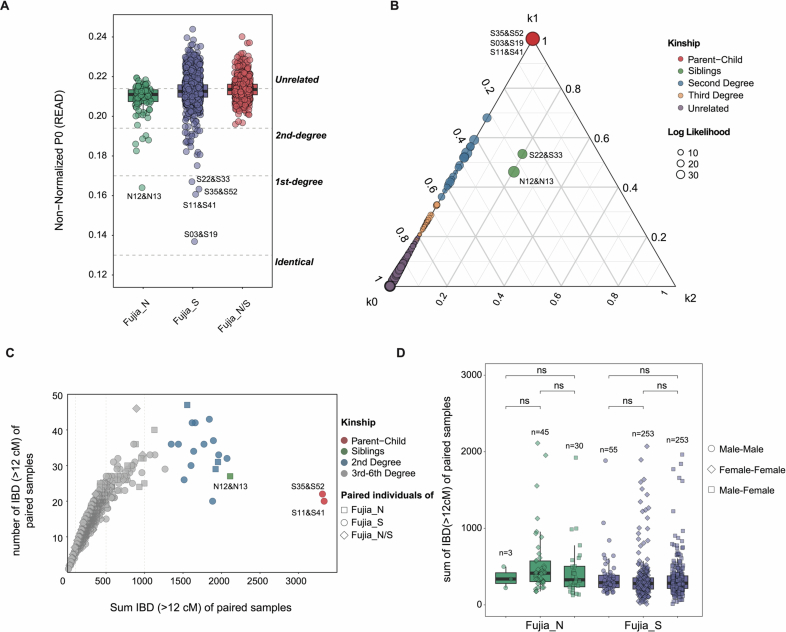
Estimation of biological relatedness for Fujia individuals. **A**, Pairwise differences (P0) calculated using the READ and plotted for both inter- and intra-cemetery comparisons. A dashed line represents the threshold for unrelatedness, determined by the median value from all pairwise comparisons, including inter-cemetery groups (Fujia_N, n = 105 pairs; Fujia_S, n = 1035 pairs) and intra-cemetery group (Fujia_N/S, n = 690 pairs). This line represents the normalization baseline and establishes cutoffs for classifying degrees of relatedness into second-degree, first-degree, and identical categories. **B**, The ternary plot of KIN results depicting the IBD sharing probabilities among paired individuals. The parameters *k*_*0*_, *k*_*1*_, and *k*_*2*_ indicate the probabilities of sharing zero, one, or two chromosomes IBD at any given locus, respectively. This plot facilitates the identification of various kinship levels, including parent-child, sibling, second-degree, and third-degree relationships, based on their respective IBD sharing states. The log likelihood values are represented by the sizes of the circles, with values greater than 1 being considered reliable. **C**, The IBD sharing analysis plots pairs of individuals based on their shared long blocks of IBD (>12 cM). These pairings are color-coded by degrees of relatedness and represented using different shapes to distinguish interactions within and between burials, indicating extensive kinship both within individual cemeteries and across different burial sites. **D**, The distribution of IBD sharing among paired individuals, classified by genetic sex within cemeteries, shows no significant differences in the amount of shared IBD between male-male and female-female pairs. The number of paired individuals analyzed from each group are labelled at the top of their boxplots. Boxplots display the median (centre line), first and third quartiles (lower/upper hinges), and whiskers extending up to 1.5 × IQR from the hinges. The term “ns” indicates that the differences are not statistically significant, as determined by a two-sided *t*-test.

**Extended Data Fig. 3:**
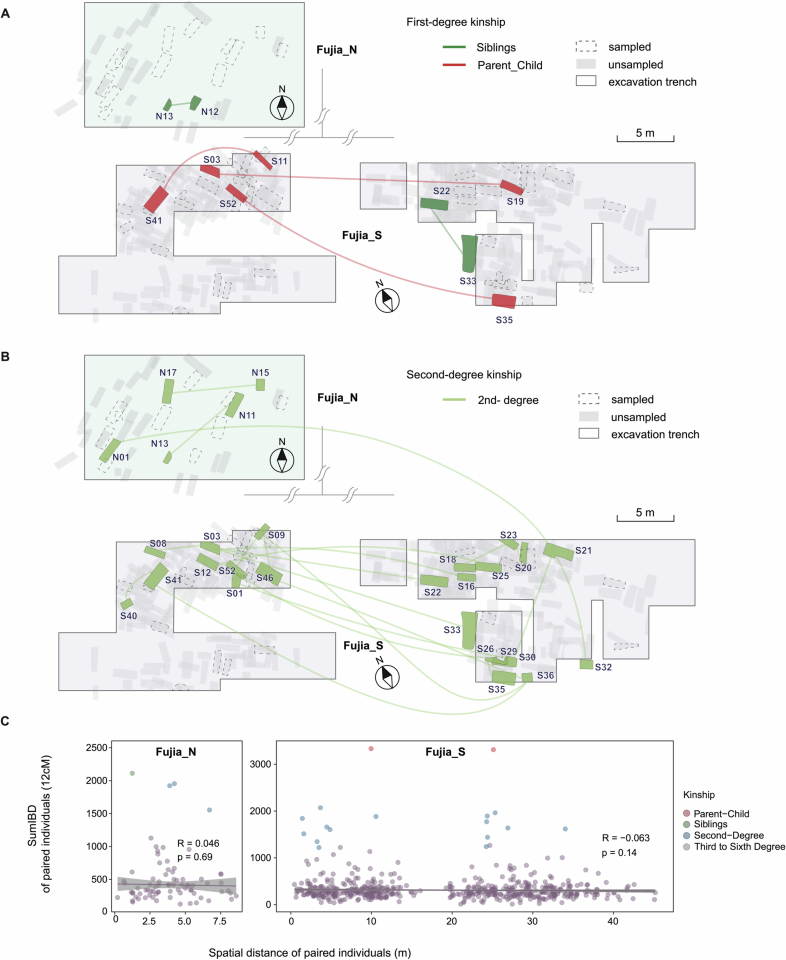
The spatial distribution of paired close relatives within cemeteries. A pair of related individuals is linked by a connecting line. **A**, Spatial distribution of paired first degree relatives, classified as parent-child and siblings. **B**, Spatial distribution of second-degree relatives. **C**, Two-sided Spearman correlation of burial spatial distance and genetic affinity (shared IBD) among paired individuals. The coefficients and *p*-values were indicated on the plot. The results revealed that neither the Fujia_N nor the Fujia_S populations exhibited a significant correlation between burial spatial distance and genetic affinity.

To understand the kin relationships among these individuals, we conducted comprehensive analyses of genetic relatedness among the 60 individuals in this study. We utilized READ^30^ to identify first-degree genetic relationships (Extended Data Fig. 2a, Supplementary Fig. 9, Supplementary Data 4 and Supplementary Note 4), KIN^31^ to detect up to third-degree relatives and to distinguish between siblings and parent–child relationships (Fig. 2b and Extended Data Fig. 2b), and ancIBD^32^ to uncover biological relatedness up to the sixth degree (Fig. 2b and Extended Data Fig. 2c). Our analysis identified that individuals N01 and S32 were avuncular relatives (Fig. 2d and Supplementary Note 4), as well as two pairs of third-degree relationships, N01 and S51, and N04 and S32 (Fig. 2b). These findings further confirmed that such close genetic relatedness did not break the adherence to matrilineal burial practices. In addition, all individuals meeting quality criteria from Fujia_S and Fujia_N exhibited a dense distribution of shared identity-by-descent (IBD) segments (>12 cM), with cumulative lengths ranging from 28 to 975 cM (median, 255 cM; Fig. 2e, Extended Data Fig. 3c and Supplementary Fig. 10). More than 96.8% (1,047 of 1,081) of individual pairs shared IBD segments exceeding 100 cM (Supplementary Data 4), indicating familial connections spanning fourth- to sixth-degree relationships. Despite comprehensive genetic relatedness between individuals from the two segregated cemeteries and the presence of the secondary burials (Supplementary Data 1), which indicates that the initial burials were not necessarily at the Fujia site, the burial practice based on maternal lineage remained strictly consistent.

The intra-cemetery comparison exhibited longer lengths of IBD and more second-degree relatives than the inter-cemetery comparison (Fig. 2b,f), indicating a closer biological relatedness between individuals within the same maternal lineage than that between two different maternal lineages. Considering the large amount of human remains and the extended utilization duration (more than 200 years) of the Fujia cemeteries, along with the uniformity of the matrilineages and high diversity of the patrilineages, it can be inferred that the Fujia cemeteries were intended for maternal clans (or matrilineal descent groups) rather than matrilineal extended families or household groups. Skeletal age estimation of the Fujia individuals also revealed that both teenage and adult male individuals were exclusively buried in the cemetery of their native matrilines (Supplementary Data 1). This practice aligns with the common norms of a matrilineal society^33^, which does not mirror patrilineal and patrilocal systems, in which female individuals are typically buried alongside their male spouses. Additionally, the burial locations of individuals within each cemetery do not correlate with genetic distance. Even first-degree relatives in the same cemetery may not necessarily be placed nearby (Extended Data Fig. 3a–c, Supplementary Fig. 11 and Supplementary Data 5). These results indicate that burial location is primarily determined by matriclan affiliation, rather than factors such as sex, age, kinship or genetic bottlenecks. Therefore, we argue that the distinct maternal lineages between Fujia_S and Fujia_N reflect an intentional organizational structure based on matriclan identity.

## High local endogamy in Fujia society

**Fig. 3:**
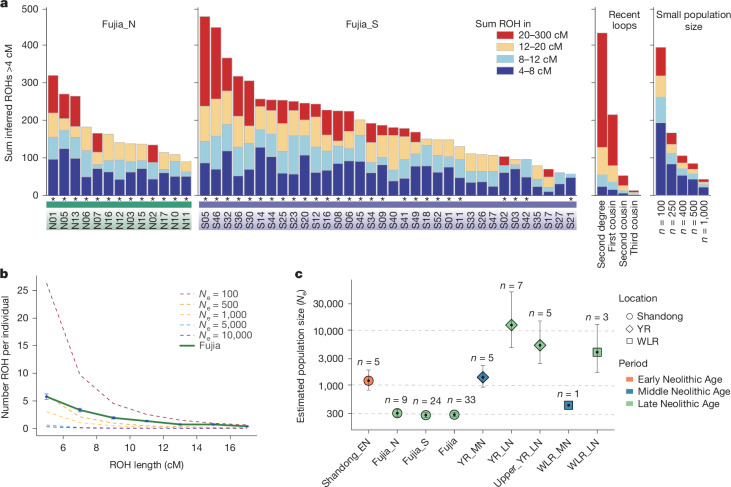
ROH and estimation of effective population size for Fujia individuals. Individuals overlapping with the 1240k panel and possessing more than 200,000 single nucleotide polymorphisms were included. **a**, Inferred ROH for Fujia_N and Fujia_S individuals are presented. Expectations are provided for inbred individuals ranging from second-degree relatives to first–third cousins, as well as for individuals from small populations of varying sizes. The ROH length distribution for Fujia corresponds to that expected from a small population size. Asterisks denote individuals with short ROH segments (<8 cM) whose cumulative length exceeds 40 cM. **b**, The average number of ROH segments across various length categories in Fujia individuals (*n* = 33). Fourteen highly consanguineous individuals were excluded on the basis of the presence of ROH segments longer than 20 cM and totalling more than 50 cM. Vertical error bars represent ±1 s.e.m. for each length bin (2 cM). The dashed lines illustrate the expected distribution of ROH for various effective population sizes (*N*_e_). **c**, Estimated effective population size of Fujia and other ancient East Asian populations, indicating Fujia had a small population size. Black points represent maximum-likelihood estimates, with vertical error bars indicating 95% confidence intervals. Sample sizes (number of individuals analysed per site) are annotated above each corresponding point. YR, Yellow River; WLR, West Liao River; EN, Early Neolithic; MN, Middle Neolithic; LN, Late Neolithic.

**Extended Data Fig. 4:**
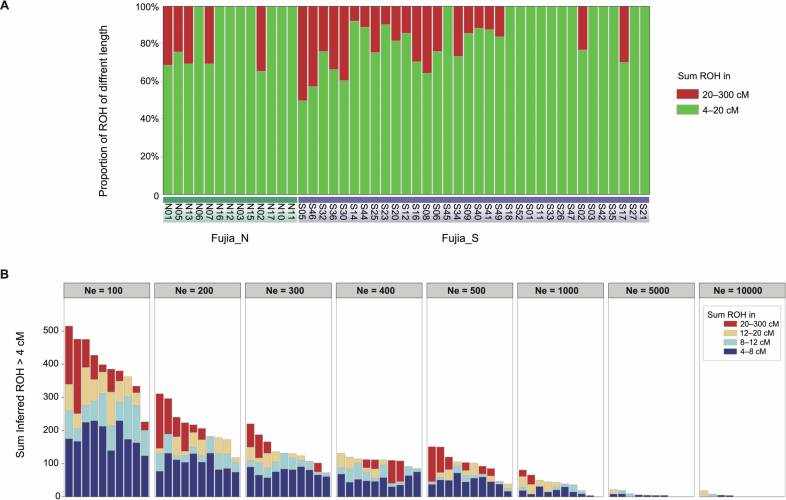
ROH patterns observed in Fujia individuals. **A**, The distribution of ROH by individual within the Fujia populations was examined. Short and medium-sized ROH segments (4–20 cM) constituted a significant proportion of the total ROH length. **B**, The simulated distribution of ROH for different effective population sizes (*Ne*). The ROH patterns in Fujia closely resembled those simulated under conditions of small *Ne*.

Further investigation of 47 individuals (34 of Fujia_S and 13 of Fujia_N) revealed a notably high prevalence of runs of homozygosity (ROH; Fig. 3a). Specifically, all individuals exhibited short ROH (<8 cM), with the sum length ranging from 10 to 127 cM, and 40 (85%) individuals had a sum length of greater than 40 cM. The total length of ROH is mostly contributed by the short- and medium-sized ROH (4–20 cM; Extended Data Fig. 4a). This ROH distribution resembled the anticipated patterns of populations with limited size in the simulated data (Fig. 3b and Extended Data Fig. 4b). Our results indicate high background relatedness probably caused by frequent mating within the Fujia community owing to limited gene pools, which also lead to a greatly reduced effective population size (*N*_e_) estimation^34,35^. Compared with that of other ancient East Asian populations, the clearly smaller *N*_e_ for the Fujia population (around 200–400 for both Fujia_S and Fujia_N; Fig. 3c) seems not to be attributed to geographical isolation as copious contemporary archaeological sites have been found in the Shandong area (Fig. 1b), but is primarily due to the reduced gene pool caused by the practice of endogamy within the community. For individuals with genomes with blocks of long ROH (>20 cM), only 4 of 47 showed more than 100 cM, indicating the low-frequency existence of consanguinity, such as mating of first cousins or above (Supplementary Data 6). Apart from these four individuals, around 47% (*n* = 22) exhibited at least one ROH segment exceeding 20 cM, probably caused by the frequent intermarriage between individuals with relatively distant familial relationships, such as second or third cousins. Coupling this with the distribution of short ROH (Supplementary Fig. 12), consanguinity is not the preferred marriage pattern but is an inevitable occurrence in a small endogamous community.

## Fujia subsistence strategies and mobility

**Fig. 4:**
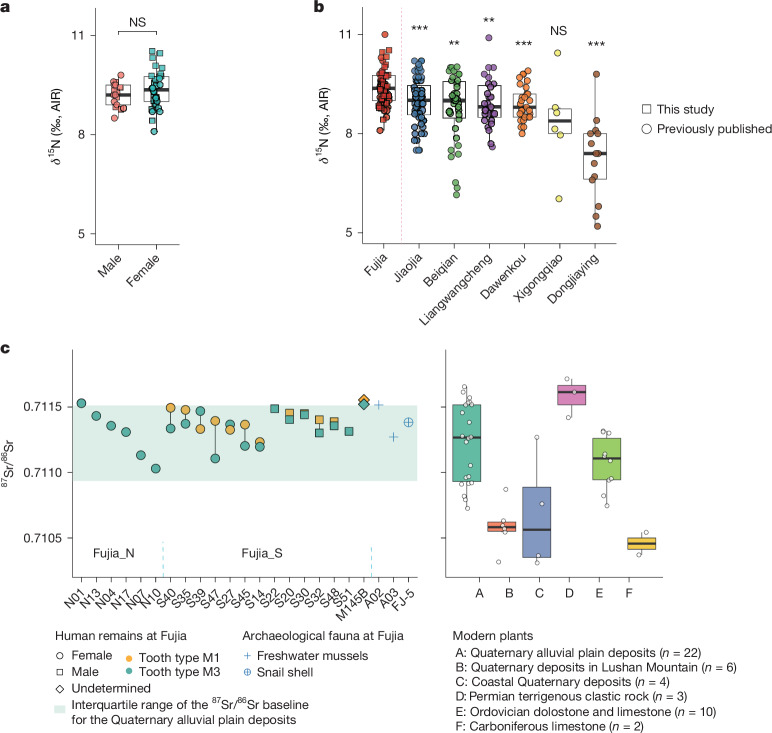
Stable isotopic analyses of the Fujia individuals. **a**, *δ*^15^N values in bone and dentine collagen of Fujia male (*n* = 17) and female (*n* = 34) individuals, showing no significant difference by two-sided *t*-test, and no adjustments were made for multiple comparisons. AIR, atmospheric air standard. **b**, *δ*^15^N value comparisons between Fujia and Dawenkou culture sites. Two-sided *t*-tests compared Fujia (*n* = 51) with six contemporary sites: Jiaojia (*n* = 60, *P* = 5.9 × 10^−4^), Beiqian (*n* = 38, *P* = 0.0012), Liangwangcheng (*n* = 27, *P* = 0.0076), Dawenkou (*n* = 29, *P* = 2.9 × 10^−4^), Xigongqiao (*n* = 7, *P* = 0.13) and Dongjiaying (*n* = 14, *P* = 1.9 × 10^−5^; Supplementary Data 2). ***P value < 0.001; **P value < 0.01; NS, not significant; no adjustments were made for multiple comparisons. **c**, ^87^Sr/^86^Sr ratio in tooth enamel for Fujia individuals (*n* = 20) compared with values from wild plants (*n* = 53) collected across different lithological units and archaeological animal samples (*n* = 3). For box plots in all panels, centre lines denote median values; whiskers denote 1.5× the interquartile range; dots represent observed values.

The *δ*^13^C values of 52 individuals from Fujia range from −9.3‰ to −6.6‰, with a mean of −7.9 ± 0.5‰ (29 from this study and 23 from a previous study^36^), indicating a predominantly C_4_-based diet (Supplementary Fig. 14 and Supplementary Data 2). The primary dietary staples probably included foxtail millet (*Setaria italica*), broomcorn millet (*Panicum miliaceum*) and millet-fed pigs^36^, with archaeobotanical evidence suggesting a greater reliance on foxtail millet-based food^27,37^. The *δ*^15^N values of humans range from 8.1‰ to 10.5‰ (*n* = 52; averaging 9.3 ± 0.5‰), with an offset of about 2.3‰ from Fujia pigs (7.0 ± 0.6‰)^36^, indicating a diet incorporating C_4_-fed animal protein. No significant differences in *δ*^13^C or *δ*^15^N values were observed between male and female individuals at Fujia or other middle or late Dawenkou sites (Fig. 4a, Supplementary Fig. 15 and Supplementary Note 5), suggesting uniform dietary practices across genetic sexes. Notably, human *δ*^15^N values at Fujia are significantly higher than those at other Dawenkou sites (Fig. 4b). This elevation may reflect the influence of saline soil conditions associated with the site’s coastal setting during the late Dawenkou period^36,38,39^ and/or dietary intake of marine and freshwater resources, as evidenced by the presence of marine molluscs at nearby sites (for example, Wucun)^40^ and freshwater mussels at Fujia and other Dawenkou sites (see Supplementary Note 5 for further discussion). These findings collectively suggest that Fujia inhabitants engaged in millet-based farming and animal husbandry, with probable access to marine and/or freshwater resources.

Strontium isotope analyses of 20 individuals, including 12 with paired molars^41^, reveal a remarkably narrow ^87^Sr/^86^Sr range of 0.711030 to 0.711554 (Fig. 4c and Supplementary Data 2). This range falls well within the baseline range of the Quaternary alluvial plain deposits where the Fujia site is located, as established by local wild plants, and is distinct from those of other geological units in the region (Fig. 4c), indicating that all Fujia individuals probably resided within the Quaternary alluvial plain deposits surrounding the site. Although some individuals exhibited ^87^Sr/^86^Sr values overlapping with those of Ordovician dolostone and limestone in the nearby mountain, the minimal variation in oxygen isotopes among the Fujia population (1 s.d. < 1‰, *n* = 23)^42^ further indicates that these individuals did not originate from high-altitude regions but rather shared a common water source (see Supplementary Note 5 for details). At a more localized scale, within the Quaternary alluvial plain deposits, all individuals’ ^87^Sr/^86^Sr values fall within the ranges of plants collected within a 10-km radius of the archaeological site (≈0.7107–0.71153) and align closely with the ^87^Sr/^86^Sr values of a snail shell and freshwater mussels from Fujia (Fig. 4c), suggesting that the Fujia population probably had limited residential mobility. These findings further support the idea that the Fujia population may have been geographically constricted, with no discernible influence of long-distance exogamy, in contrast to other contemporaneous or later Neolithic groups in China^43^. The homogeneous oxygen isotope signatures^42^ and the narrowest dietary niche breadth among Dawenkou populations further indicate a localized scope of activities and relatively uniform livelihood practices within the Fujia community. Genetic analyses corroborate these findings, indicating endogamy practices and a probably cohesive community (Supplementary Note 5). Although male individuals generally exhibited a slightly narrower ^87^Sr/^86^Sr range compared to female individuals, no statistically significant difference was found (*P* value = 0.149) using a Mann–Whitney test.

## Discussion

More than a century ago, sociologists such as Johann Jakob Bachofen^44^, Lewis Henry Morgan^45^ and Friedrich Engels^46^ proposed that ancient societies were generally matrilineal, evolving into patrilineal structures with the advent of private property. Since the twentieth century, the hypothesis of an early matrilineal stage in human history has been vigorously debated by social anthropologists^47^. Some have argued that the idea of a matrilineal stage was based on Greek myths and deductions^12^, and others have pointed out a lack of support for matrilineal societies in the study of other primates as well as a lack of direct archaeological evidence^48^. This topic can now be revisited using more persuasive scientific approaches, combining new data and methods, especially those relating to ancient genomes.

Our results indicate that the Fujia community was probably organized around matrilineal principles. This interpretation is supported by the strong correspondence between genetic features and cemetery boundaries. Individuals buried in the same cemetery not only shared the same mitochondrial haplogroup but, in most cases, also possessed identical consensus mtDNA sequences, indicating close maternal ancestry and probably a shared matrilineal identity. The high ratio (60.9%, 392 of 644 pairs) of the observed second- to sixth-degree kinship connections between these two cemeteries also suggests a long-term coexistence of the two cemeteries, which have been used for more than approximately 250 years. These patterns parallel those found in modern matrilineal societies, which typically consist of multiple matrilineal clans (such as the duolocal Mosuo, matrilocal Lahu and Tlingit^14,15,17,18^), in which individuals maintain lifelong affiliation with their natal matriclan and are buried accordingly. However, the relationship between residence and burial at Fujia remains unresolved, as post-marital residence cannot be definitively deduced from burial practices or genetic data alone. Ethnographic studies reveal diverse residence patterns (for example, duolocal, matrilocal or avunculocal) across matrilineal societies, although individuals universally retain natal clan membership and burial ties^33^. The cemeteries at Fujia may therefore represent a Neolithic matrilineal organization that, although potentially varying in structure from those of later periods, remains fundamentally in shared maternal descent.

ROH analysis revealed a high rate of endogamy within the Fujia population, without a preference for consanguineous unions. This pattern differs from that of contemporaneous patrilineal societies in Neolithic Europe, where female exogamy was more commonly practiced^6,49,50^. This unique social organization is further emphasized by minimal ROH observed in other Neolithic East Asian populations, such as those from the Yellow River and West Liao River regions, underscoring the distinctiveness of Fujia’s social organization. The observed endogamy may reflect a pattern in which male individuals married women from nearby communities to maintain their authority within the kin group. Similar practices have been documented in modern Southeast Asian matrilineal and matrilocal societies, in which high levels of village endogamy and low genetic diversity are common^51^. Despite general population growth from the Middle to Late Neolithic in areas such as the Yellow River basin, West Liao River^52^ and the Shandong Peninsula, Fujia maintained a relatively small effective population size (Fig. 3c), even compared to Early Neolithic Shandong populations.

Nevertheless, genetic patterns alone do not provide conclusive evidence for matrilineal descent. For instance, the Samaritans, who follow patrilineal inheritance rules, exhibit very low mtDNA diversity^53^, demonstrating that limited maternal variation can also arise in non-matrilineal contexts. Conversely, maternal descent transmission has been observed in matrilineal groups with high mtDNA haplogroup diversity^54^. An alternative explanation to matrilineal membership recognition could involve a strong maternal founder effect or a demographic bottleneck in both ancestral populations to account for the observed mtDNA uniformity, followed by strong social constraints on female mobility combined with male-mediated gene flow, wherein women are prevented from marrying outside their group, whereas men move between communities. Under this scenario, distinct mitochondrial gene pools could be maintained without institutionalized matrilineal inheritance. Considering the broad archaeological context of Fujia, especially the spatial proximity and extensive genetic relatedness between burial groups, an interpretation involving persistent matrilineal social organization may be more coherent.

The Fujia site is located in a region that stretches from the northern foothills of the Tai-Yi Mountains to the southern coast of Bohai Bay. This area contains numerous contemporaneous Dawenkou culture sites that share closely related archaeological characteristics and are collectively referred to as the Wucun type of Dawenkou culture^55^. Common traits across these sites include painted and relatively coarse pottery, subsistence strategies based on millet farming with marine resource supplementation, and generally small to medium settlement sizes with limited evidence of social hierarchy (Supplementary Note 1, Supplementary Fig. 1 and Supplementary Table 1). Sites such as Wucun have also exhibited high grave densities^40^, similar to those observed in Fujia. This suggests that Fujia reflects a broader regional pattern rather than a unique or isolated case.

At the same time, Fujia existed during a period of increasing social complexity in Late Neolithic China^56,57^. The late Dawenkou culture, to which Fujia belongs, located mainly in Shandong, Henan and Anhui province, experienced a population peak^52^ and exhibited many large settlements as regional centres with elite tombs that are seen as different polities, featuring high-value funerary goods such as jades and ivories, suggesting a network of elite exchanges across regions^58,59^. However, Fujia contrasts with other late Dawenkou regional centres such as Jiaojia and Gangshang in its lower wealth, low levels of social stratification (Supplementary Notes 1 and 6, Supplementary Fig. 16 and Supplementary Data 7), and possibly low population density (estimated from settlement sizes; Jiaojiao and Gangshang both exceed 80 hectares in area, which is twice the size of Fujia). Fujia’s social organization and subsistence patterns differ from those of highly stratified societies^60^, and suggest that its social dynamics resemble those of modern matrilineal societies with a lower degree of heritable resources and private properties.

Our integrated analysis of ancient DNA, stable isotope data and archaeological evidence suggests the presence of a distinct social structure at Fujia that aligns with characteristics commonly associated with matrilineal organization. These findings lend support to the hypothesis that early matrilineal systems could emerge and persist in communities without strong mechanisms for wealth accumulation. The Fujia case provides insights into how such structures may have developed under specific social and environmental conditions during the Neolithic transition towards more complex societies in East Asia. Ongoing in-depth research in ancient DNA and archaeology will further elucidate the structure and dynamics of matrilineal kinship and social organization in early human societies.

## Methods

### Ancient sample preparation and DNA extraction

Samples from 66 individuals of the Fujia archaeological site were obtained from Shandong Provincial Institute of Cultural Relics and Archaeology. Priority was given to petrous bones (*n* = 43), with tooth samples (*n* = 23) processed when petrous bones were unavailable. Ancient DNA laboratory work was conducted in specialized facilities at the School of Archaeology and Museology and the Biomedical Pioneering Innovation Center at Peking University. The processing involved cutting petrous bones into pieces from the denser region around the cochlea^63^. These pieces were then subjected to a cleaning protocol that included washing with 2% sodium hypochlorite, multiple ethanol washes, and UV irradiation on each side to remove surface contamination. Similarly, tooth samples were washed with 2% sodium hypochlorite, mechanically abraded, ethanol-washed, and UV-irradiated, and then drilled into fine powder. For DNA extraction, petrous bone pieces and tooth powder of 50–100 mg were digested in a 1 ml solution of 0.45 M EDTA and 0.25 mg ml^−1^ proteinase K, following a published protocol with minor modifications^64^. DNA purification was performed using large-volume spin columns from a High Pure Viral Nucleic Acid Large Volume Kit (Roche), yielding 60 µl of DNA extract per sample.

### DNA library construction and sequencing

DNA extracts were converted into double-stranded libraries using the NEBNext Ultra II DNA Library Prep Kit for Illumina (NEB), incorporating several modifications from the standard protocol to optimize for ancient DNA characteristics. Initially, the DNA underwent treatment with USER enzyme at 37 °C for 50 min, followed by UGI enzyme treatment at the same temperature for 30 min in end-repair buffer. After these treatments, End-prep enzyme was added to the mixture. Adaptor ligation was performed using Y-shaped adaptors at a concentration of 0.1 μM to increase efficiency. This was followed by an initial amplification step using KAPA Uracil Hot-start HiFi Ready Mix. The libraries were then purified using VAHTS DNA Clean beads (Vazyme) to remove adaptor dimers. The resulting short-length libraries underwent a second round of amplification using double-indexed P5–P7 primers and Q5 Master Mix (New England Biolabs) to generate full-length libraries. The completed libraries were quantified using fragment analysis and sequenced on an Illumina NovaSeq platform using paired-end 2 × 150 cycle runs. Base calling and filtering of exact index sequences were conducted using CASAVA 1.8.2 software. Low-quality reads, defined as having an average or 50% Phred quality value ≤ 20, were discarded to ensure the integrity of the sequencing data.

### Ancient DNA data processing and quality control

The raw sequenced read data (fastq files) were processed through an nf-core/eager v2.3.2 pipeline^65^ (https://nf-co.re/eager). We clipped the Illumina sequencing adaptors by AdapterRemoval v2.3.1 (ref. ^66^). We mapped merged reads to the human reference genome (hs37d5; GRCh37 with decoy sequences) using the bwa v0.7.17 aln/samse alignment algorithm^67^ with the parameters -n and -l set to 0.01 and 1,024, respectively. The reads with phred mapping quality of less than 30 were then discarded using -q (q30-reads) in Samtools v1.9 (ref. ^68^). We removed PCR duplicates using DeDup v0.12.2 (ref. ^69^). To minimize the impact of post-mortem DNA damage on genotyping, we prepared additional ‘trimmed’ BAM files by soft masking 2 base pairs on both ends of each read using the trimbam function on bamUtils v1.0.13 (ref. ^70^) on the basis of the DNA misincorporation pattern of each library. For the single nucleotide polymorphism (SNPs) in the 1240k panel, we randomly sampled a single high-quality base (Phred-scaled base quality score 30 or higher) as pseudodiploid genotypes using the PileupCaller program (https://github.com/stschiff/sequenceTools) with the --randomHaploid flag by randomly choosing one high-quality base (phred base quality score ≥30) on the 1240k panel^71,72^.

### In-solution ancient DNA capture

Enrichment for the 1240k panel SNPs and mtDNA was conducted at the Biomedical Pioneering Innovation Center, Peking University in Beijing. The 1240k DNA probes were synthesized on the basis of previously published datasets^73^ and subsequently reverse-transcribed into RNA probes. These RNA probes, along with human mtDNA probes obtained from iGeneTech (TargetSeq One Kit, lot TC1NTC2N), were used for targeted enrichment. The mtDNA probes were diluted 100-fold and combined with the 1240k probe for simultaneous enrichment. A total of 500 ng of DNA library was used for each capturing process. The DNA libraries were first mixed with blocking oligonucleotides (Hyb Human Block and Universal Block from iGeneTech, designed for Illumina TruSeq Libraries), followed by adding biotinylated RNA probes (1240k and mtDNA), RNase block and hybridization buffer. The mixture was denatured at 85 °C for 5 min and hybridized at 50 °C for 24 h. The hybridized products were then incubated with streptavidin-capped beads for 30 min at room temperature. Unbound DNA was removed using Wash Buffer 1 at room temperature for 15 min, followed by three high-temperature washes with TargetSeq One Wash buffer at 50 °C for 10 min each. The beads were then washed with ethanol, dried and resuspended in Q5 High-Fidelity Polymerase Master Mix for amplification using P5 and P7 primers. The amplification thermal profile was as follows: initial denaturation at 98 °C for 3 min, followed by 14 cycles of 98 °C for 15 s, 60 °C for 30 s, and 72 °C for 30 s, with a final extension at 72 °C for 5 min. The post-capture libraries were purified using VAHTS DNA Clean beads and quantified using a Qubit fluorometer (Thermo Fisher). The libraries, once quantified by fragment analysis, were sequenced on an Illumina NextSeq 500 platform using paired-end 2 × 75 cycles.

### Ancient DNA authentication

Various methods were used to authenticate the ancient genomes sourced from Fujia. Initially, we examined patterns of post-mortem chemical modifications characteristic of ancient DNA using the software mapDamage v2.0.6 (ref. ^74^). Subsequently, mitochondrial contamination rates were calculated for each individual using Schmutzi v1.5.1 (ref. ^75^). Additionally, the nuclear genome contamination rate in male individuals was determined by analysing X chromosome data with ANGSD v0.910 (ref. ^76^). As male individuals possess only one X chromosome, discrepancies between bases aligned to the same polymorphic position, exceeding the threshold of sequencing error, were interpreted as indicators of contamination.

### Sex determination and uniparental genetic marker analyses

The genetic sex of the individuals from whom our ancient samples were obtained was determined by analysing the ratio of X- and Y-chromosome coverage relative to autosomes^77^. Consensus mtDNA sequences were called using Schmutzi v1.5.1 (ref. ^75^), and the resulting FASTA files were utilized to determine mtDNA haplogroups using Haplogrep3 (ref. ^78^) of PhyloTree 17 (revised Cambridge reference sequence). Y-chromosome haplogroups were determined on the basis of uniquely mapped reads on the human reference genome hg38 using the bwa mem command. We collected all of the available 32,656 SNPs from an up-to-date Y-chromosome phylogenetic tree (http://yoogene.com/sourse/) and called each sample on these SNPs. Each haplogroup on the Y tree has a set of theoretical nucleotide genotypes calculated from the ancestral and derivative states of SNPs. For each sequenced sample, all SNP calls (with at least 1× coverage and no heterozygosity) were compared to the theoretical nucleotide calls of all the haplogroups, and the haplogroup with highest similarity score was assigned to the sample. Owing to low sequencing coverage of ancient DNA, only a small proportion of haplogroup-defining SNPs could be called. Therefore, we excluded samples in which fewer than 1,000 Y-chromosome SNPs were unambiguously called, as well as those with potential contamination. The retained male samples (3 from Fujia_N and 10 from Fujia_S) were then categorized through their called haplogroups, with samples potentially belonging to the same paternal clan classified in the same category (Supplementary Data 1).

### Kinship estimation

To estimate kinship among ancient individuals from the Fujia site, we used three established methods optimized for ancient DNA. We utilized READ v1.0 (ref. ^30^) to identify first- and second-degree relatives among the Fujia individuals. It calculates the proportion of non-matching alleles (P0) within 1-megabase non-overlapping windows, on the basis of pseudo-haploid genotypes from the 1240k SNP set. We assumed that the median of the average P0 (0.214 for Fujia) across all pairs represents unrelated individuals. Identical twins were defined as having a normalized average P0 of less than 0.625 (non-normalized P0 < 0.136), first-degree relatives were defined as having a normalized average P0 of less than 0.8125 (non-normalized P0 < 0.17), and second-degree relatives were defined as having a normalized average P0 of less than 0.90625 (non-normalized P0 < 0.194). We used a hidden Markov model-based approach (KIN v3.1.3)^31^ to detect patterns of shared IBD states along genomes, accurately identifying up to third-degree relatives even with low coverage (as little as 0.05×). It differentiates relationships by analysing three IBD states: zero (k0), one (k1) or two (k2) chromosomes shared IBD at any given position. For example, the IBD sharing probabilities for siblings are (0.25, 0.5, 0.25). Using genome windows of 10 Mb, the software infers IBD across a range of kinship categories and assigns each pair to the model with the maximum likelihood. We consider results with a log likelihood ratio of greater than 1.0 as reliable. We used ancIBD v0.3a1 (ref. ^32^) to analyse kinship in ancient populations up to the sixth degree of relatedness. It extends the analysis beyond close kinship to explore genetic associations across an entire population through more distant relationships.

### Imputation and shared IBD estimation

The genetic data were imputed utilizing the GLIMPSE2 software^79^, which is well suited for handling low-coverage data (<0.5×). Following the standard procedures outlined in the GLIMPSE2 tutorials (https://odelaneau.github.io/GLIMPSE/docs/tutorials), the software was used to impute the genetic data. To perform imputation, phased haplotypes from the 1000 Genomes Project were used as a reference panel (http://ftp.1000genomes.ebi.ac.uk/vol1/ftp/release/20130502/). The imputed and phased data were then used as the input to the software ancIBD (v0.3a1; https://pypi.org/project/ancIBD/)^32^. Following the software’s recommended guidelines, we screened all pairs among the Fujia individuals (*n* = 54), ensuring each pair had at least 270,000 SNPs covered. This screening was aimed at detecting long IBD segments (>8 cM) using the default settings provided by ancIBD^32^. Summary statistics were recorded for each pair for which IBD was detected.

### Analysis of ROH and population size

We conducted an analysis of ROH and estimated the effective population size (*N*_e_) using the hapROH software (v0.64)^34^. This involved examining data from 47 individuals (13 from Fujia_N and 34 from Fujia_S), each with coverage of more than 200,000 SNPs using the 1240k capture assay. The 1000 Genomes haplotypes served as our reference panel, and we adhered to default parameter settings. For each individual, we calculated various ROH summary statistics. These included the number and cumulative length of ROH segments exceeding specific thresholds (4, 8, 12 and 20 cM), as well as the maximum length of any single ROH segment. To distinguish between endogamy and inbreeding effects within our dataset, we used a cutoff: a sum of ROH segments greater than 20 cM indicated potential inbreeding. Our focus was primarily on ROH segments ranging from 4 to 20 cM to provide reliable *N*_e_ estimates. We excluded individuals exhibiting extensive inbreeding signs, specifically those with more than 40 cM of ROH longer than 20 cM. The estimation of *N*_e_ was performed using the MLE_ROH_Ne function in hapROH, with adjustments made according to the recommended guidelines to ensure accurate settings were applied. To simulate typical distributions of ROH lengths in panmictic populations with various effective population sizes, we used coalescent simulation with the software msprime^80^. We defined ROH as chromosome regions delimited by two recombination events that coalesce into a common ancestor when simulating the coalescence of two haplotypes. Simulations were conducted on all autosomes, with each chromosome simulated separately. Chromosome lengths were determined on the basis of the map difference between the first and last 1240k SNP on each autosome. For each effective population size (*N*_e_) value ranging from 100 to 10,000, ROH were simulated for 1,000 independent diploid individuals. Subsequently, the average total length and count values were calculated for each ROH length bin, as defined in the analysis of the Fujia data.

### Principal component analysis

We performed principal component analysis using smartpca v18140 (ref. ^81^). The analysis included a set of 266 present-day East Asian individuals from the HumanOrigins dataset. We used the options lsqproject: YES’and shrinkmode: YES.

### *F* statistics

We used outgroup *f*_3_ statistics^82,83^ to obtain a measurement of genetic relationship between two populations since their divergence from an African outgroup. We calculated *f*_4_ statistics with the f4mode: YES function in admixtools^82^. *f*_3_ and *f*_4_ statistics were calculated using qp3Pop v435 and qpDstat v755 in the admixtools package.

### Stable isotope analysis

The collection of 53 modern wild-plant samples was carried out on the basis of lithology and rock age data from a 1:200,000 high-resolution geological map^84^ (Supplementary Fig. 13). To ensure representative coverage, denser sampling was conducted in larger geological units (for example, Quaternary alluvial plain deposits; Supplementary Data 2). All sampling locations were carefully selected to minimize potential anthropogenic influence. Specifically, most plant samples collected from mountainous or elevated areas were located at least 500 m away from modern settlements, major roads and cultivated farmland. These areas are sparsely inhabited and largely undisturbed. For the area surrounding the Fujia site, plant samples were taken from within the Fujia Archaeological Park, a protected zone where urban and agricultural development has been prohibited for more than a decade. All sampled plants were naturally growing species, with no evidence of landscaping or artificial soil treatment. In other regions, samples were collected at a minimum distance of 50 m from roads, residential areas and cultivated fields. Although we made every effort to avoid anthropogenic contamination during sampling, we acknowledge that it remains difficult to fully assess the extent of modern influence on the collected plant samples. This limitation has been explicitly discussed in Supplementary Note 5. To further validate the local strontium isotope baseline, we also analysed one snail shell and two freshwater mussel shells excavated from the Fujia site, as these organisms reflect Sr isotopes directly from their environments.

For strontium isotope analysis, we collected 32 tooth samples from all available individuals (*n* = 20), with 12 individuals providing paired samples, primarily the first permanent molars (M1) and the third permanent molars (M3), formed at 0–3 and 9–14 years of age, respectively^41^. For carbon and nitrogen isotope analysis, we sampled petrous bones from 28 individuals and dentine material (M2 and M3) from 6 individuals.

Collagen extraction followed the protocol described in the literature^85^. Approximately 500 mg of bone material was sampled from each individual. The samples were demineralized in 0.5 M HCl for several weeks until CO_2_ evolution ceased, then treated with 1% NaOH to remove humic acid contamination, and re-acidified in 0.5 M HCl. The samples were then rinsed with Milli-Q water before gelatinization in pH 3 HCl at 90 °C. Insoluble fractions were filtered, and the purified collagen solution was frozen and freeze-dried. *δ*^13^C and *δ*^15^N measurements were conducted at the School of Archaeology and Museology, Peking University, using an isotope ratio mass spectrometer (IsoPrime-100 IRMS) coupled with an elemental analyser (Vario Pyro Cube). *δ*^13^C and *δ*^15^N values are reported in per mille (‰) relative to the Vienna PeeDee Belemnite and AIR standards, respectively. Analytical error, based on repeated measurements of IAEA600 and USG41 standards, was better than 0.2‰ for both *δ*^13^C and *δ*^15^N values. Isotopic analyses, including those to determine the percentage of C, the percentage of N and C/N ratios, followed established protocols^86^ and quality control parameters. Of the 34 samples analysed, 29 contained well-preserved collagen (Supplementary Data 2).

Plant samples were rinsed three times with Milli-Q water, then dried and ashed in a muffle furnace at 800 °C for 12 h. To further minimize surface contamination of the archaeological shells, we first removed the outer layers and surface deposits using a dental drill, followed by five rinses with Milli-Q water. The shell samples were then ultrasonically cleaned in ultrapure acetone for 10 min to remove any organic residues. For human and animal samples, approximately 10 mg of tooth enamel and shell powder was drilled from each individual using a handheld drill with a diamond-tipped burr. All plant, tooth enamel and shell samples were digested in 2 ml of 65% HNO_3_. The solution was evaporated to dryness and re-dissolved in 1 ml of 3.5 N HNO_3_ at 120 °C for 1 h. Strontium was separated using 100–150-µm Sr-spec resin (Triskem International). All ^87^Sr/^86^Sr ratios were determined using a Neptune Plus multi-collector inductively coupled plasma mass spectrometer (Thermo Fisher Scientific) at Beijing Createch Testing Technology, alongside the international standard NIST SRM-987. An ^88^Sr/^86^Sr ratio of 8.375209 was used to correct for mass fractionation. The standard SRM-987 yielded an average ^87^Sr/^86^Sr ratio of 0.710247 ± 0.000014 (2σ, *n* = 67), consistent with the standard value of 0.710250.

### Reporting summary

Further information on research design is available in the Nature Portfolio Reporting Summary linked to this article.

## Online content

Any methods, additional references, Nature Portfolio reporting summaries, source data, extended data, supplementary information, acknowledgements, peer review information; details of author contributions and competing interests; and statements of data and code availability are available at 10.1038/s41586-025-09103-x.

## Supplementary information

Supplementary Information Supplementary Notes 1–6, References, Tables 1–4 and Figs. 1–16.

Reporting Summary

Supplementary Data 1 Sample information.

Supplementary Data 2 Stable Isotope Analysis.

Supplementary Data 3 mtDNA sequences.

Supplementary Data 4 Kinship estimation for Fujia individuals.

Supplementary Data 5 ROH of Fujia individuals.

Supplementary Data 6 Burial distance for Fujia cemeteries.

Supplementary Data 7 Grave values of Fujia and other sites relevant to this study.

## Data Availability

Raw and alignment data have been deposited at the Genome Sequence Archive in the National Genomics Data Center under the accession number HRA007862 (https://ngdc.cncb.ac.cn/gsa-human/browse/HRA007862). The previously reported ancient DNA datasets used in this study are available in the Allen Ancient DNA Resource v54.1 (https://reich.hms.harvard.edu/allen-ancient-dna-resource-aadr-downloadable-genotypes-present-day-and-ancient-dna-data). The dataset used for ^14^C radiocarbon date calibration is IntCal20 (http://intcal.org). The reference panel used for the Y-haplogroup assignment is based on a current Y-chromosome phylogenetic tree (http://yoogene.com/sourse/). The Genome Reference Consortium Human Build 37 (GRCh37) is available through the National Center for Biotechnology Information under the BioProject accession number PRJNA31257. The revised Cambridge reference sequence is available through the National Center for Biotechnology Information under the Reference Sequence accession number NC_012920.1. The base map for Fig. 1b was obtained from the Word Terrain Base domain map dataset (https://www.arcgis.com/home/item.html?id=c61ad8ab017d49e1a82f580ee1298931) and created with ArcGIS v10.2.

## Code availability

The analyses presented in this study rely on publicly available software, which is cited in the main text and Supplementary Information.
