# Supplementary material for: Ancient DNA reveals a two-clanned matrilineal community in Neolithic China
Source: Nature. 2025 Jun 4;643(8074):1304–11. doi: 10.1038/s41586-025-09103-x (PMC12310535; doi:10.1038/s41586-025-09103-x)
Supplement: Supplementary file 1 — Supplementary Notes 1–6, References, Tables 1–4 and Figs. 1–16. [file 41586_2025_9103_MOESM1_ESM.pdf]

nature portfolio

https://doi.org/10.1038/s41586-025-09103-x

Supplementary information

Ancient DNA reveals a two-clanned matrilineal community in Neolithic China

In the format provided by the authors and unedited

Supplementary Materials: Ancient DNA Reveals a Two-Clanned Matrilineal Community in Neolithic China

This PDF file includes:

Supplementary Text

Supplementary Note 1 Regional overview and archaeological context of Fujia
Supplementary Note 2 Radiocarbon dating and duration of Fujia site use
Supplementary Note 3 Population genetics of the Fujia individuals
Supplementary Note 4 Biological relatedness of the Fujia individuals
Supplementary Note 5 Stable isotope data analysis
Supplementary Note 6 Grave value of the Dawenkou cultural archaeological sites

References

Other supplementary materials for this manuscript include the following:

Supplementary Data S1 Sample information (.xlsx)
Supplementary Data S2 Stable Isotope Analysis (.xlsx)
Supplementary Data S3 Mitochondrial DNA sequences (.xlsx)
Supplementary Data S4 Kinship estimation for Fujia individuals (.xlsx)
Supplementary Data S5 Runs of Homogeneity (ROH) of Fujia individuals (.xlsx)
Supplementary Data S6 Burial distance for Fujia cemeteries (.xlsx)
Supplementary Data S7 Grave values of Fujia and other sites relevant to this study (.xlsx).

Supplementary Note 1. Regional overview and archaeological context of Fujia.

The Dawenkou culture, which flourished from approximately 5800 to 4400 years ago in the Haidai region of China, primarily in the eastern province of Shandong, but also extending into Anhui, Henan, and Jiangsu, represents a significant epoch in Neolithic China[1]. It was first discovered in 1959 at the Dawenkou site in Tai'an, Shandong, and has since been recognized for its considerable contribution to the understanding of ancient Chinese civilization. This culture, thriving in the fertile basins of the Wen and Si Rivers, following the Houli and Beixin cultures[2] and preceding the Longshan and Yueshi cultures. The archaeological significance of the Dawenkou culture is underscored by its long duration and wide influence, which have been focal points of academic research, exploring its socio-economic systems, burial customs, social structures, settlement patterns, and the chronological phases of its development[3].

The Dawenkou culture is divided by researchers into early, middle, and late phases, each characterized by distinct advancements in subsistence strategies and societal organization. Initially, the economy was predominantly based on hunting and gathering, especially in the eastern coastal areas and inland lake regions of the Jiaodong Peninsula. However, there was a gradual shift towards agriculture, with the cultivation of dry crops like millets becoming increasingly prominent. By the middle to late Dawenkou period, agriculture experienced unprecedented growth, marked by the introduction of rice from the south, which became a staple in some regions, highlighting the culture's adaptability and agricultural innovation[4].

Artisanship within the Dawenkou culture reached impressive levels, particularly in the production of jade, bone, ivory crafts, and pottery[4]. Jade artifacts, primarily flake-shaped, evolved in technique from line cutting to flake cutting, indicating technological progress and changing cultural practices. The function of jade also transitioned from decorative to both ritualistic and decorative, reflecting its growing significance in Dawenkou society. Bone and ivory craftsmanship, utilizing materials from large animals like elephants, deer, wild boars, alligator, turtle, and demonstrated advanced techniques such as cutting, grinding, carving, openwork, and drilling. Pottery, too, was a significant aspect of their material culture, with the invention of the fast wheel during the middle to late Dawenkou period marking a peak in craftsmanship. The pottery often featured intricate tri-colored designs of white, black, and red, achieving high artistic standards.

Socially and structurally, the early Dawenkou culture exhibited initial signs of internal differentiation within settlement clusters, leading to the emergence of central and ordinary settlements. This trend continued, with the formation of a pyramid-shaped, three-tier settlement structure by the middle to late phases, which included large central settlements often fortified with defensive moats, indicating a more complex societal organization. The differentiation in burial customs paralleled these social changes; early graves were relatively egalitarian, but later graves began to emphasize quantity and quality of grave goods, including multi-layered coffins, suggesting increasing social stratification.

The diet of the Dawenkou people was well-adapted to their environment, with a heavy reliance on foxtail and broomcorn millets in later stages. Archaeological findings have

revealed sophisticated storage systems capable of holding large quantities of grain, indicating a successful agricultural base. Additionally, the culture's domestication of animals such as dogs and pigs, played a crucial role in their economy, with pigs being particularly significant as evidenced by their frequent occurrence in archaeological sites and burials[5,6]. Additionally, the Dawenkou practiced dental ablation and cranial deformation, practices that disappeared in China by the Bronze Age[7,8].

In summary, the Dawenkou culture was a dynamically evolving society with significant advancements in agriculture, craftsmanship, and social structure. Its contributions to the development of social complexity, technological innovation, and cultural practices in Neolithic China provide invaluable insights into the prehistoric development of the region, bridging earlier cultures with those that followed and shaping the trajectory of Chinese civilization. This culture's extensive influence and technological innovations, such as trepanation and symbols that may represent some of the earliest forms of writing[9], highlight its sophisticated and complex nature, making it a subject of enduring interest and importance in the archaeological study of ancient East Asia.

This paper discusses the Fujia site[10], an important archaeological site from the Dawenkou period, dating back 4,750–4,500 years ago. Located in Shandong province, north of Tai-yi Mountain near the southern part of the Bohai Sea, the site covers approximately 37 hectares. The area, where the Fujia site is located, is widely distributed with Dawenkou cultures sites. Spanning approximately 8,000 square kilometers, this region is home to a total of 96 Dawenkou sites (Figure S1A). The distances between these sites range from 0.23 to 13.25 kilometers. While some smaller sites may be part of larger ones, the average distance between sites is 4.11 kilometers, and they generally exhibit an even distribution pattern. According to the records of the Third National Cultural Heritage Census, the area of each site varies from 0.12 to 40 hectares (Table S1). Again, some smaller sites might be integral parts of larger complexes. Compared to Dawenkou sites in other parts of Shandong, most of the sites in this area are small to medium sized. The Fujia site stands out as a relatively large site within this local cluster but is only of medium size when considered among all Dawenkou sites across the Shandong region (Figure S1B)

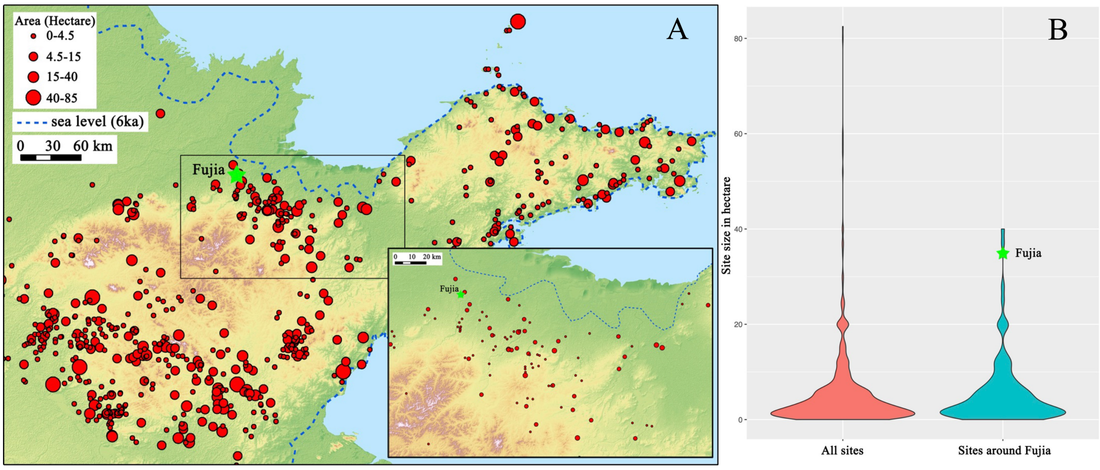

Figure S1. Dawenkou archaeological sites. A. Distribution of Dawenkou

archaeological sites in Shandong Province (n=724). The map was plotted based on an open source GDEMV2 30M by ArcGIS. The dataset is provided by Geospatial Data Cloud site, Computer Network Information Center, Chinese Academy of Sciences. (http://www.gscloud.cn). B. Comparison of the Fujia archaeological site with other Dawenkou sites (n=102) in terms of size.

Table S1 Dawenkou sites around Fujia in northern Shandong

ID	Name	Longitude	Latitude	Area (Hectare)
1	Fengjia	119.829246	36.981046	7.5
2	Sanbuli	119.679963	36.986639	3.75
3	Hancun	119.564041	36.739051	28
4	Xuwang	118.21232	36.957109	7.5
5	Guojiaqiao	118.193503	36.924923	2
6	Tonglin	118.252744	36.904435	1
7	Donggu	118.408792	36.902724	0.25
8	Cuijia	118.392795	36.882132	0.64
9	Dafuguan	118.383921	36.857647	0.2
10	Yafu	118.401222	36.858187	0.81
11	Xuejia	118.398085	36.850721	0.5
12	Xuejiaxi	118.397294	36.844714	3.75
13	Houlibei	118.398322	36.842362	0.5
14	Houlinan	118.397766	36.837928	3
15	Qianli	118.396356	36.831984	1
16	Beiling	118.375116	37.133945	8
17	Wucun	118.451407	37.081495	6
**18**	**Fujia**	**118.408407**	**37.046364**	**37**
19	Rongzhuang	118.452328	36.985381	12
20	Xiwoshi	118.43687	36.952326	6
21	Zhongyang	119.718462	37.046594	3
22	Chenjia	118.963981	36.776192	6
23	Xujia	119.078968	36.681109	0.75
24	Qianbuxia	119.389159	36.747098	1.5
25	Lujiakou	119.144839	36.8199369	4
26	Majia	119.005898	36.802296	1.5
27	Shizihang	119.311083	36.803148	14
28	Yuanshang	119.34814	36.687517	5.5
29	Beiliu	119.314954	36.618154	10.5
30	Taoyuan	118.589672	36.725933	3.75
31	Xuwang	118.524909	36.941746	2
32	Mazhuang	118.54948	36.916682	3
33	Nanduan	118.510068	36.895688	2
34	Zhuma	118.567826	36.850377	13
35	Sunban	118.593358	36.809402	1.3
36	Hougudian	118.52555	36.776695	4.5
37	Shijia	118.601708	36.745983	3.6
38	Dachen	118.583062	36.809634	5
39	Xishi	118.67625	36.727218	5.8
40	Dongshi	118.694646	36.72535	6
41	Beixiguan	118.47206	36.695753	2
42	Xiaquan	118.446958	36.676262	8.75

43	Wuli	118.426176	36.68147	0.8
44	Yupanshi	118.676765	36.729282	19.5
45	Daguanying	118.564907	36.647707	0.08
46	Qianliangtai	119.475442	36.297466	4.3
47	Songjiapo	119.408965	36.224136	10
48	Chengzi	119.50478	36.293319	2
49	Xuejia	118.79154	36.705716	5
50	Houtong	118.739007	36.979197	2
51	Wangzhuang	118.674717	37.014375	6
52	Zhaowangpu	118.766762	36.901029	8.5
53	Zhaojiashi	118.799803	36.882372	4.4
54	Donghoujia	118.742063	36.836059	2
55	Xihoujia	118.738325	36.832807	1.38
56	Daziliu	118.707606	36.830795	3
57	Houhuying	118.733848	36.82835	25
58	Huoshanbu	118.770564	36.833734	5
59	Zhujiayu	118.690197	36.802046	2.5
60	Xiaoyangjia	118.657362	36.798054	2
61	Sanyuanwang	118.668666	36.787191	3.75
62	Qingtianhu	118.830113	36.770362	12
63	Anjia	118.725331	36.735501	6.2
64	Quanzilangjun	119.048258	36.386004	6
65	Xinanzhuang	119.154003	36.327222	12
66	Tianjialou	118.836191	36.302979	12
67	Jingzhi	119.391691	36.311664	0.0053
68	Nanlu	118.802938	36.2612	3
69	Laoduoyu	119.075092	36.226303	20
70	Dongjia	119.530546	36.753923	40
71	Anjia	118.725835	36.425148	0.8
72	Dongjiagou	118.569834	36.582553	2
73	Xizhufeng	118.5203	36.472443	10
74	Ganjiagou	118.81685	36.447029	2
75	Miaoshan	118.779917	36.406063	8
76	Qianyuliu	118.871911	36.746371	0.5
77	Weijiazhuang	118.941276	36.722421	20
78	Guojia	118.843349	36.699341	1.5
79	Donglishan	118.855894	36.69465	2
80	Xiaolijia	118.798942	36.699121	1.5
81	Guoqidianzi	118.889542	36.672787	1
82	Lijialao	118.809458	36.647664	0.5
83	Yangxu	118.850456	36.634961	1.5
84	Nangao	118.773138	36.630719	1.5
85	Linjiahe	118.816922	36.625424	1.5
86	Huangcun	118.772972	36.618586	1.2
87	Gengjia	118.774618	36.599985	4
88	Zoujiazhuang	118.773889	36.581587	20
89	Jia	119.064489	36.581777	0.15
90	Dahexi	119.098545	36.551339	1
91	Xiji	118.933085	36.524874	8
92	Chengjiahe	118.923702	36.522516	0.12
93	Gaijia	118.909207	36.522619	2

94	Xili	118.786621	36.451985	4.5
95	Zhaike	119.055894	36.463801	6
96	Xiaojiazhuang	118.98137	36.4005	2

Situated on elevated ground surrounded by depressions, the Fujia site yielded Dawenkou cultural graves, wells, house foundations, and storage pits. Notably, it had lots of large, orderly arranged wells, a rarity among Dawenkou cultural sites. The abundant artifacts recovered, primarily pottery, exhibit distinct regional characteristics and provide valuable data for the study of early cultures in northern Shandong. Physical anthropology measurements on human skeletons from Fujia_S provide basic knowledge on burial practices at Fujia. Of the 295 individuals, 58% are identified as male and 42% as female. Among all the burials, 35% of the 355 individuals are identified as secondary burials, with joint burials involving multiple skeletons accounting for 12%. Based on age estimations of the 143 skeletons excavated in 1995, the even distribution of all age ranges (mean age = 23.4 years) indicates no age bias in Fujia's burial practices (Figure S2).

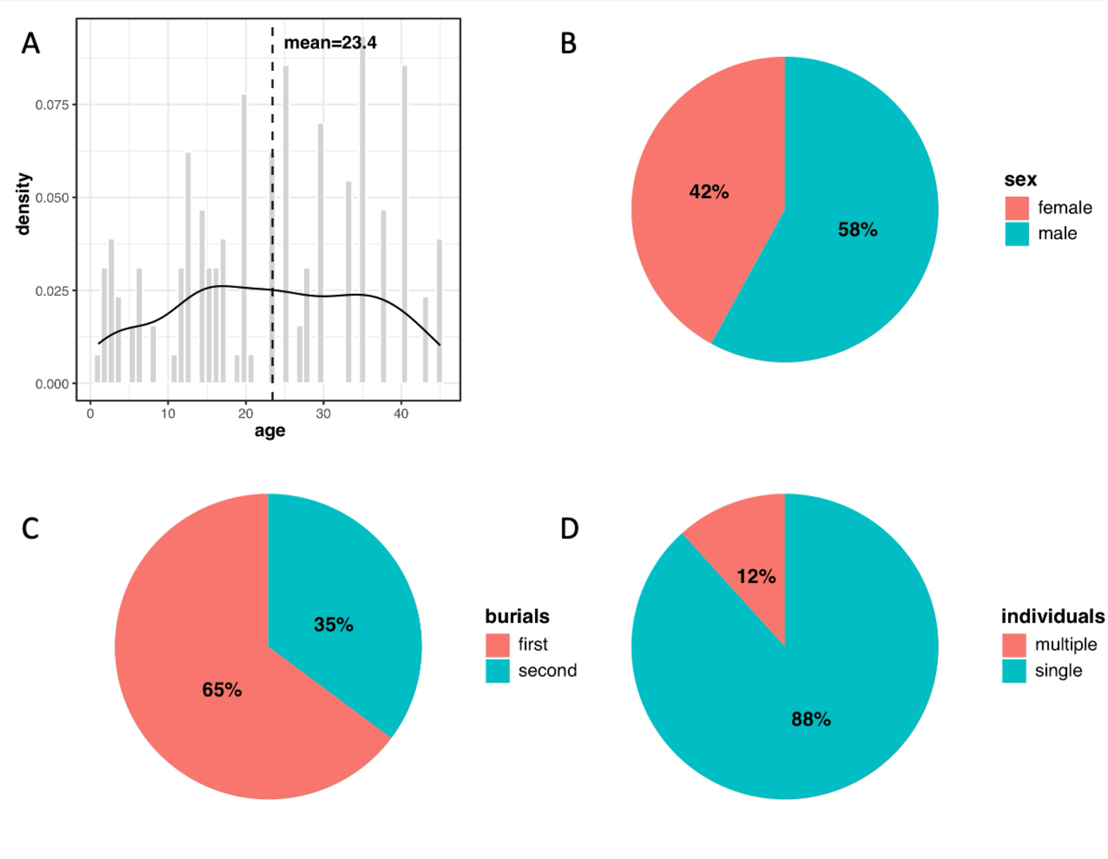

Figure S2. Physical anthropological analyses of the Fujia_S individuals. A. Individual age distribution of death in Fujia_S (n=143). B. Pie chart indicating the proportion of males and females in Fujia_S (n=295). C. Pie chart showing the proportion of first and second burials for Fujia_S (n=355). D. Pie chart showing the proportion of sing and multiple burials in Fujia_S (n=171).

Supplementary Note 2. Radiocarbon dating and duration of Fujia site use

To date the tombs and estimate the duration of the matrilineal cemeteries in Fujia, 19 newly sampled bones/teeth samples were intentionally selected for AMS dating. These included 15 samples from the southern cemetery (Fujia_S) and 4 from the northern cemetery (Fujia_N). The samples selected from each cemetery are consistent with their respective mitochondrial types. The samples were processed and analyzed at Beta Analytic Lab and the Radiocarbon Dating Lab at Peking University. In addition to 5 previously radiocarbon-dated samples from the southern cemetery[4], our dataset now includes 20 samples from the southern cemetery and 4 from the northern cemetery. The dating results are presented below (Table S2).

Table S2. Radiocarbon dating of Fujia individuals

Lab Number	Cemetery	DNA Number	Sample Number	Material	Conventional Radiocarbon Age (BP)	Calibrated date (cal BP)<br>2σ (95.4%)
Beta-661684	South	S16	M346	tooth	4010±30	(94.0%) 4530 - 4414<br>(1.4%) 4568 - 4558
Beta-661685	South	S36	M502	tooth	4160±30	(89.7%) 4828 - 4610<br>(5.7%) 4604 - 4580
Beta-661686	South	S26	M453	tooth	4120±30	(69.0%) 4725 - 4526<br>(26.4%) 4817 - 4751
Beta-661687	South	S44	T7M17	tooth	4120±30	(69.0%) 4725 - 4526<br>(26.4%) 4817 - 4751
Beta-673887	North	N13	M43	bone	4130±30	(61.1%) 4730 - 4566<br>(28.3%) 4820 – 4748<br>(6.1%) 4560 – 4530
Beta-698665	South	S47	T8M124	bone	4110±30	(55.9%) 4657 – 4522<br>(24.9%) 4816 – 4751<br>(14.6%) 4721 - 4664
Beta-698667	South	S32	M489	bone	4130±30	(61.1%) 4730 – 4566<br>(28.3%) 4820 – 4748<br>(6.1%) 4560 - 4530
Beta-698668	South	S27	M460	bone	4110±30	(55.9%) 4657 – 4522<br>(24.9%) 4816 – 4751<br>(14.6%) 4721 - 4664
Beta-698669	South	S40	T3M021	bone	4040±30	(93.8%) 4581 – 4418<br>(1.6%) 4612 - 4600
Beta-698671	South	S33	M495	bone	4150±30	(95.4%) 4825 - 4575
Beta-698672	South	S41	T6M107	bone	4130±30	(61.1%) 4730 – 4566<br>(28.3%) 4820 – 4748<br>(6.1%) 4560 - 4530
Beta-700748	North	N01	M19	bone	4110±30	(24.9%) 4816-4751<br>(14.6%) 4721-4663<br>(55.9%) 4658-4522
Beta-700749	North	N07	M17	bone	3940±30	(10.2%) 4515-4480<br>(82.8%) 4445- 4289<br>(2.5%) 4270 - 4254
Beta-700750	North	N12	M44	bone	4090±30	(18.5%) 4808- 4755<br>(6.9%) 4702 -4 670<br>(64.5%) 4652-4517<br>(5.5%) 4476-4446
BA240376	South	S12	M152	bone	4175±25	(20.8%) 4833 - 4786<br>(73.4%) 4767 - 4616<br>(1.3%) 4596 - 4586
BA240377	South	S14	M313	bone	4070±20	(5.8%) 4788 - 4764<br>(73.5%) 4619 - 4514<br>(16.2%) 4480 - 4444

BA240379	South	S42	T6M195	bone	4175±20	(20.1%) 4830 - 4791<br>(75.3%) 4764 - 4620
BA240380	South	S46	T8M104	bone	4115±25	(26.5%) 4814 - 4752<br>(14.7%) 4716 - 4665<br>(54.2%) 4656 - 4525
BA240381	South	S49	T8M44	bone	4125±20	(29.3%) 4815 - 4752<br>(18.8%) 4719 - 4664<br>(42.2%) 4656 - 4567<br>(5.1%) 4558 - 4530
A1990*	South	na	M17	bone	4175±25	(20.8%) 4833 - 4786<br>(73.4%) 4767 - 4616<br>(1.3%) 4596 - 4586
A2091*	South	na	M34	bone	4150±25	(31.1%) 4823 - 4743<br>(64.4%) 4736 - 4579
A1989*	South	na	M9	bone	4140±20	(30.4%) 4820 - 4748<br>(65.0%) 4729 - 4575
A2090*	South	na	M21	bone	4050±25	(0.9%) 4780 - 4772<br>(2.4%) 4613 - 4597<br>(92.1%) 4583 - 4422
A1991*	South	na	M32	bone	4030±20	(3.3%) 4569 - 4554<br>(92.1%) 4532 - 4421

* Samples AMS dated by Dong et al. 2021[4] and recalibrated with IntCal20.

We use the OxCal 4.4 (https://c14.arch.ox.ac.uk/oxcalhelp/hlp_contents.html) and calibrate curve IntCal20[11] to make a Bayesian estimate[12] on the burial process of the 20 tombs in the southern cemetery and 4 tombs in the northern cemetery. However, for the southern cemetery, an incessant burial model is poorly fitted when all the tombs are placed in the same phase. This indicates either not enough tomb samples were included in the model, or that a burial hiatus existed during the formation of the cemetery. We then apply a kernel density estimate (KDE) to the 20 dates in the southern cemetery. According to the means of all the 15 calibrated date probabilities in the KDE model, they can be visually divided into two groups. The first group includes 15 data points with the means of date probabilities concentrated around ~4750–4650 cal. BP. The other group, consisting of 5 data points, ranges from ~4550 to 4450 cal. BP (Figure S3).

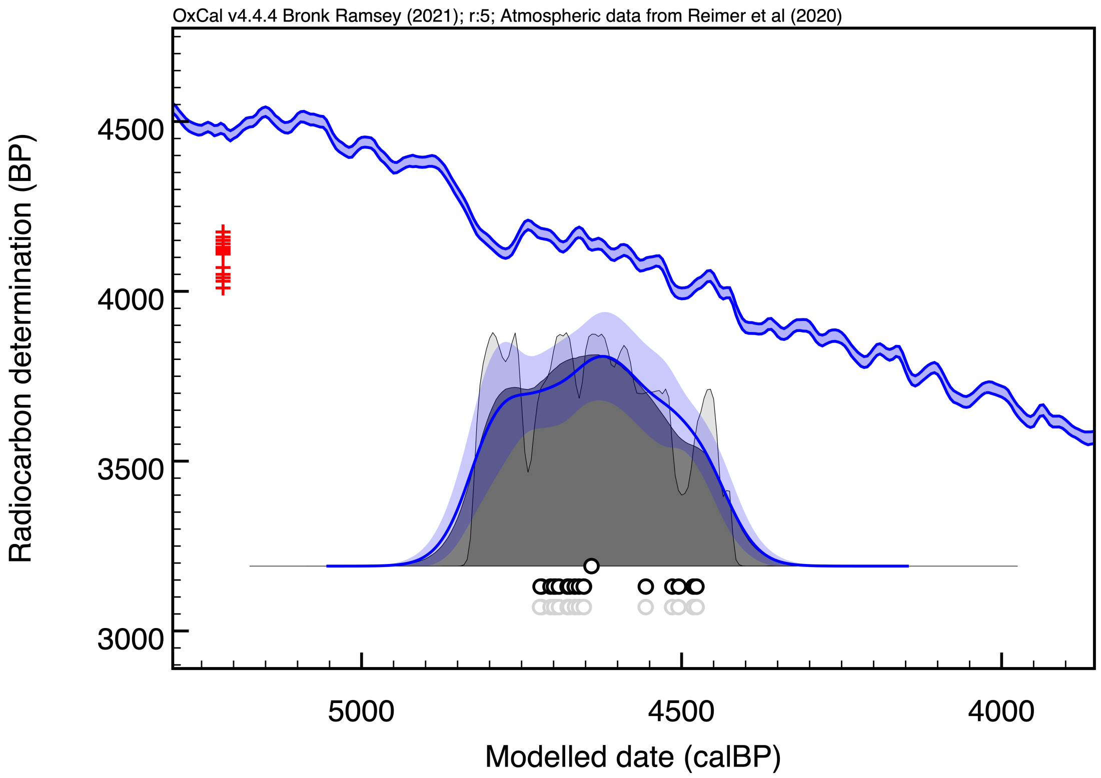

Figure S3. KDE Plot of Fujia southern cemetery dates

Based on this analysis, we create a two-stage model to place the 20 dates into two separate Phases: Phase 1 with 15 dates and Phase 2 with 5 dates, and introduce a hiatus between them. Boundaries are placed at the beginning and end to mark the start and end of the cemetery, as well as the presumed gap between the two Phases. The results are well-modeled with good agreement indices, a measure of the agreement between the model (prior) and the observational data (likelihood), with each date and the overall agreement exceeding the 60% threshold level. According to the two-stage model, the first stage (4817~4580 to 4797~4535 cal. BP, 95% probability) lasted 71~266 (mean=168, 95% probability) years whilst the second stage (4580~4449 to 4574~4442 cal. BP, 95% probability) lasted 8~118 (mean=62, 95% probability) years. The possible gap between the two stage was 0~60 (mean=21, 95% probability) years. Totally, the whole cemetery lasted for about 71~444 (mean=251, 95% probability) years. The prosperity of the cemetery was in the first stage when the tombs were intensively burred. Perhaps after a short time of hiatus, the cemetery was continuously used for much longer time but with lower frequency of inhumation (Table S3; Figure S4).

Table S3. Result of Two-stage model of Fujia southern cemetery

**Name**	**Unmodelled (BP)**			**Modelled (BP)**			**Indices A model 103.2 A overall 101.1**		

	mu	from_95_4	to_95_4	mu	from_95_4	to_95_4	Acomb	A	C
Start				4754	4858	4651			96
**Southern Cemetery phase1**									
S_M152	4719	4833	4586	4681	4817	4580		94	99.3
M17	4719	4833	4586	4681	4817	4580		94	99.3
S_T6M195	4721	4830	4620	4685	4818	4585		93.7	99.2
S_M502	4705	4828	4580	4670	4810	4573		99.5	99.5
S_M495	4697	4825	4576	4664	4808	4571		101.7	99.6
M34	4699	4823	4579	4666	4810	4573		101	99.6
M9	4692	4820	4575	4661	4805	4572		101.9	99.4
S_M489	4678	4820	4530	4652	4803	4550		104.1	99.5
S_T6M107	4678	4820	4530	4652	4803	4550		104.1	99.6
S_T8M44	4675	4815	4530	4648	4803	4552		102.3	99.6
S_M453	4667	4817	4526	4645	4797	4541		104.3	99.5
S_T7M17	4667	4817	4526	4645	4798	4541		104.3	99.7
S_T8M104	4660	4814	4525	4640	4799	4536		103.1	99.7
S_T8M124	4653	4816	4522	4638	4797	4535		103.6	99.7
S_M460	4653	4816	4522	4638	4797	4535		103.8	99.5
**Duration phase1**				**168**	71	266			98.4
**Possible Gap**				**21**	0	60			99.8
**Southern Cemetery phase2**									
S_M313	4556	4789	4444	4536	4580	4449		120.5	99.7
M21	4515	4780	4422	4523	4578	4447		103	99
S_T3M021	4505	4612	4418	4519	4575	4450		98.1	99.1
M32	4481	4569	4421	4510	4573	4445		85.5	98.9
S_M346	4477	4567	4414	4508	4574	4442		87.3	99.2
**Duration phase2**				**62**	8	118			98.7
End				4475	4560	4404			96.1

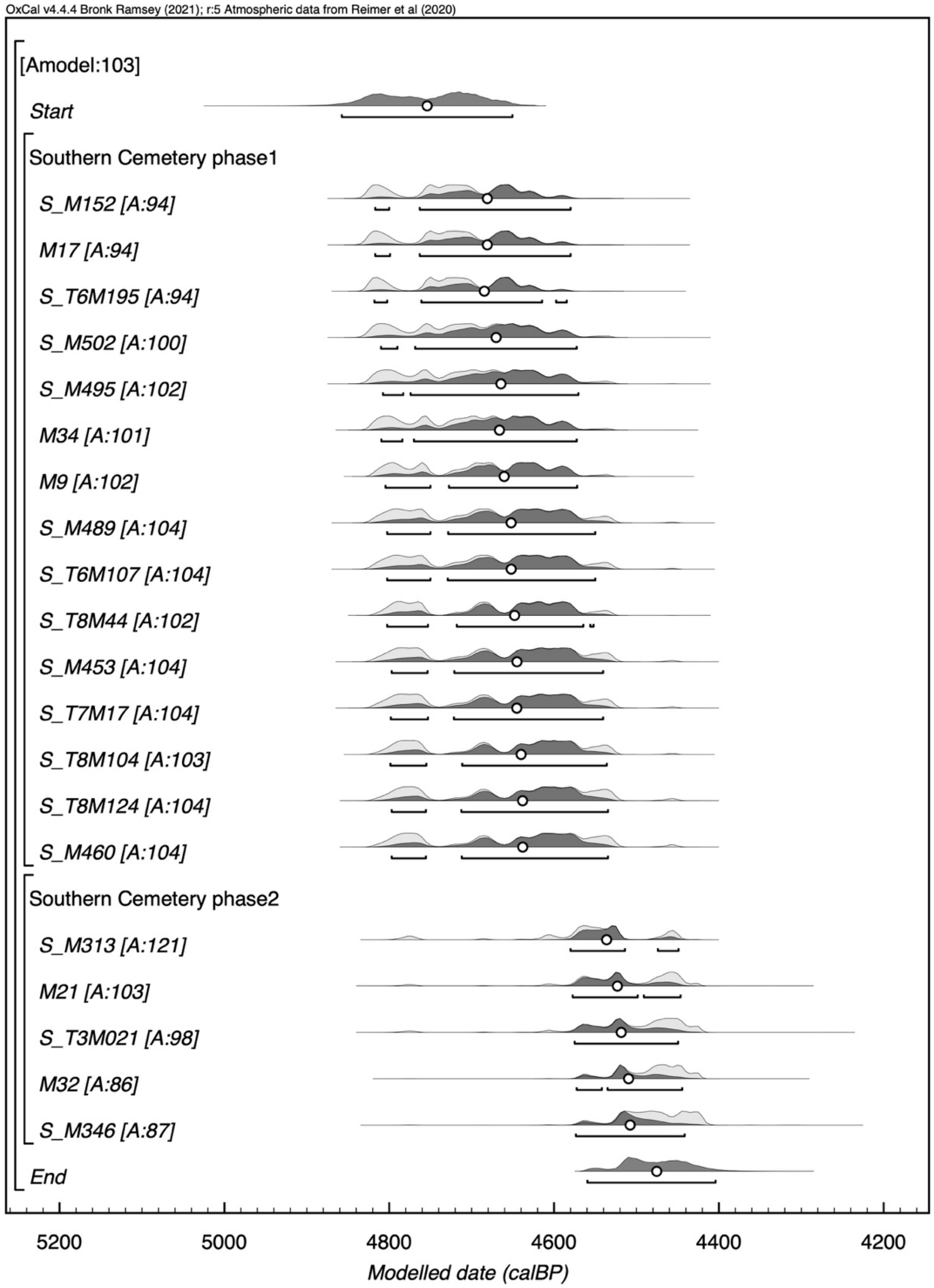

Figure S4. Two-stage model of Fujia southern cemetery

The Bayesian model was also applied to the dates of four tombs in the northern cemetery. Despite having only four dates, the cemetery was estimated to have been in use for 35 to 463 years (mean = 241 years, 95% probability) from 4807~4525 to 4522~4296 cal. BP (95% probability), which is no longer than the southern cemetery. As we know, radiocarbon dating provides dates with probability intervals rather than precise calendar dates. However, roughly speaking, the radiocarbon dates combined

with the Bayesian estimates for the tombs in Fujia suggest that both cemeteries began around 4750 BP and ended around 4500 BP, spanning about 250 years. Considering the mean death age of approximately 23.4 years, as determined by physical anthropology, both cemeteries could encompass at least ten generations (Table S4; Figure S5).

Table S4. Modeling of Fujia northern cemetery

**Name**	**Unmodelled (BP)**			**Modelled (BP)**			**Indices A model 95.2 A overall 95.3**		
	mu	from_95_4	to_95_4	mu	from_95_4	to_95_4	Acomb	A	C
Start				4794	5284	4537			96.9
**Northern Cemetery**									
N_M43	4678	4820	4530	4634	4807	4525		95.7	99.6
N_M19	4653	4816	4522	4614	4805	4453		101.3	99.7
N_M44	4617	4808	4446	4589	4798	4446		106.9	99.6
N_M17	4380	4515	4254	4429	4522	4296		87.7	99.4
**Duration**				**241**	35	463			99.4
End				4300	4523	3824			93.9

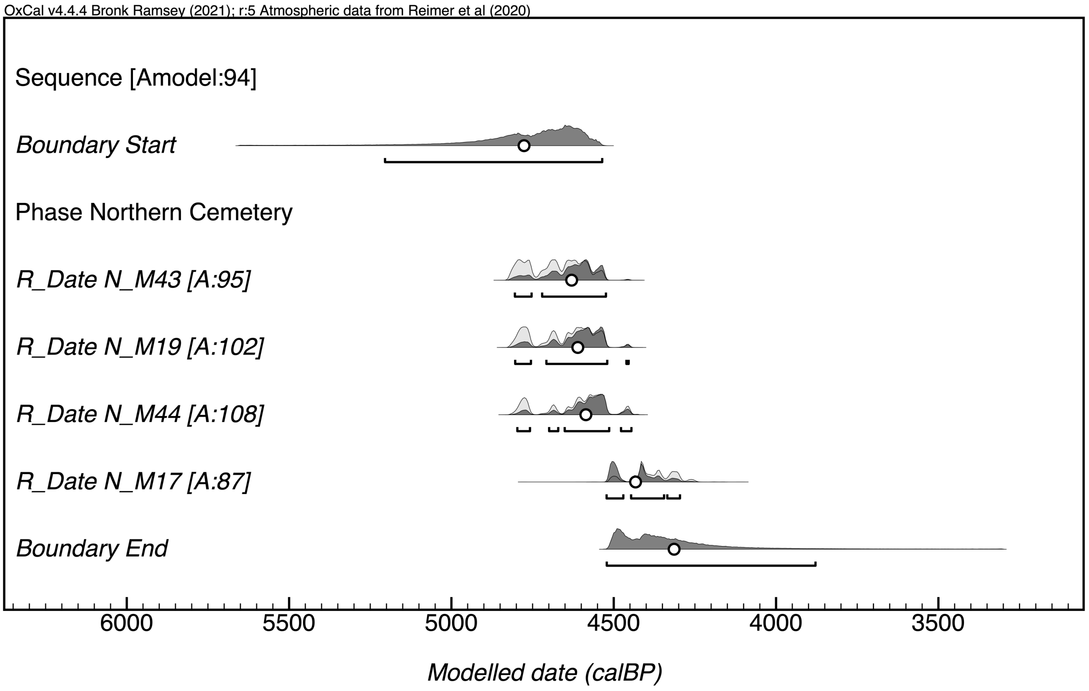

Figure S5. Modeling of Fujia northern cemetery.

Supplementary Note 3. Population genetics of the Fujia individuals

To gain insights into the genetic structure of the Fujia individuals and their genetic relationships with both earlier and later populations from Shandong and neighboring areas, we performed a PCA that included present-day East Asian populations and ancient individuals from this study, alongside relevant ancient populations documented in the literature[13–15]. The PCA results show that all Dawenkou Fujia individuals, whether from the Fujia_N or Fujia_S cemetery, form a cohesive and homogeneous cluster. These individuals are closely aligned with the Early Neolithic Houli cultural individuals from Shandong ("Shandong_EN", 6500-5500 BCE)[16], yet display a genetic shift towards the Longshan individuals from Henan ("YR_LN") and populations from southern China (Figure S6). This suggests that from the Houli to the Fujia Dawenkou culture, Neolithic populations in Shandong were strongly influenced genetically by southern China individuals. Additionally, on a broader geographical scale, the Fujia Dawenkou individuals are genetically distinct from the Yangshao culture individuals ("YR_MN") from Henan province in the Central Plains of China.

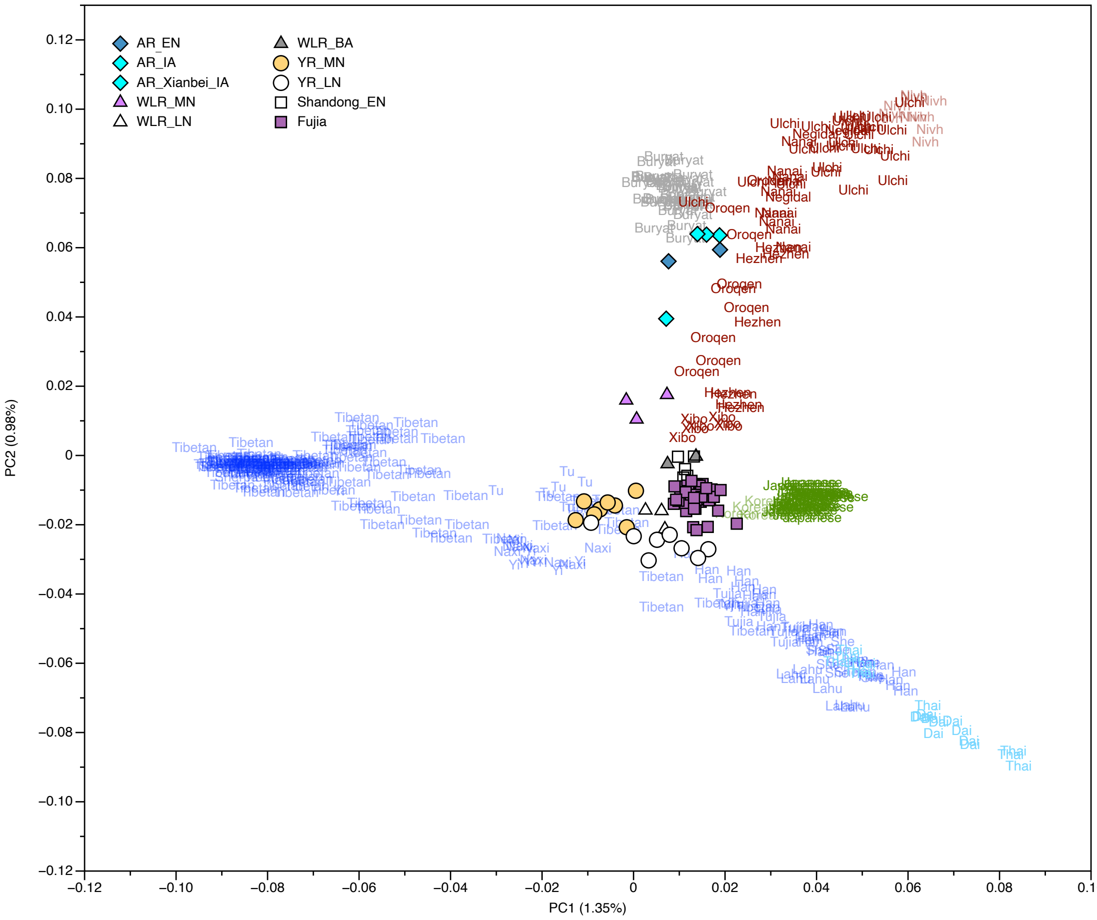

Figure S6. Principal Component Analyses of the Fujia individuals.

We excluded individuals displaying first- or second-degree genetic relationships in group-based analyses. Outgroup-f3 statistics confirm the genetic uniformity between Fujia_N and Fujia_S individuals, as they share the most alleles with each other (Figures

S7A and 7B). This finding is consistent with PCA results and symmetry tests, which also suggest that Fujia_S and Fujia_N are genetically cladal (Figures S8A and 8B). Consequently, we merged Fujia_S and Fujia_N into a single group to enhance analytical resolution. Overall, the Fujia individuals exhibit a strong genetic affinity with ancient populations from Northern China (Figures S8C–7F).

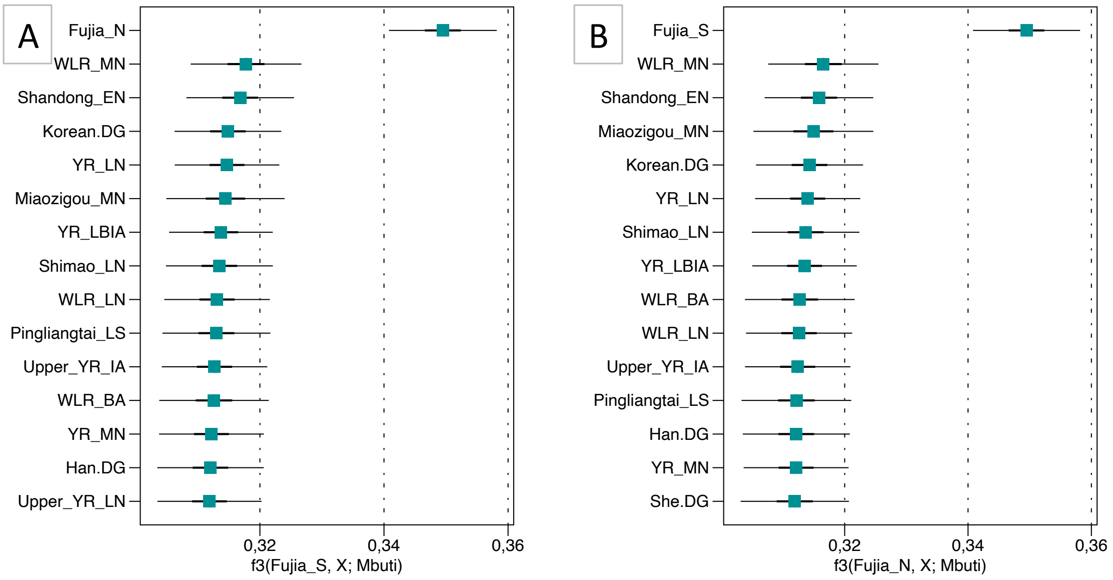

Figure S7. Outgroup-f_3 statistics for Fujia individuals. We present top 15 outgroup-f_3 signal for each population among 334 non-sub-Saharan African populations. Horizontal bars represent the point estimate ± 3 (thin) and ± 1 (thick) s.e.m, respectively, and s.e.m. are estimated using 5 cM block jackknifing. A. Allele sharing of Fujia_S to other ancient and modern world-wide populations. B. Allele sharing of Fujia_N to other ancient and modern world-wide populations.

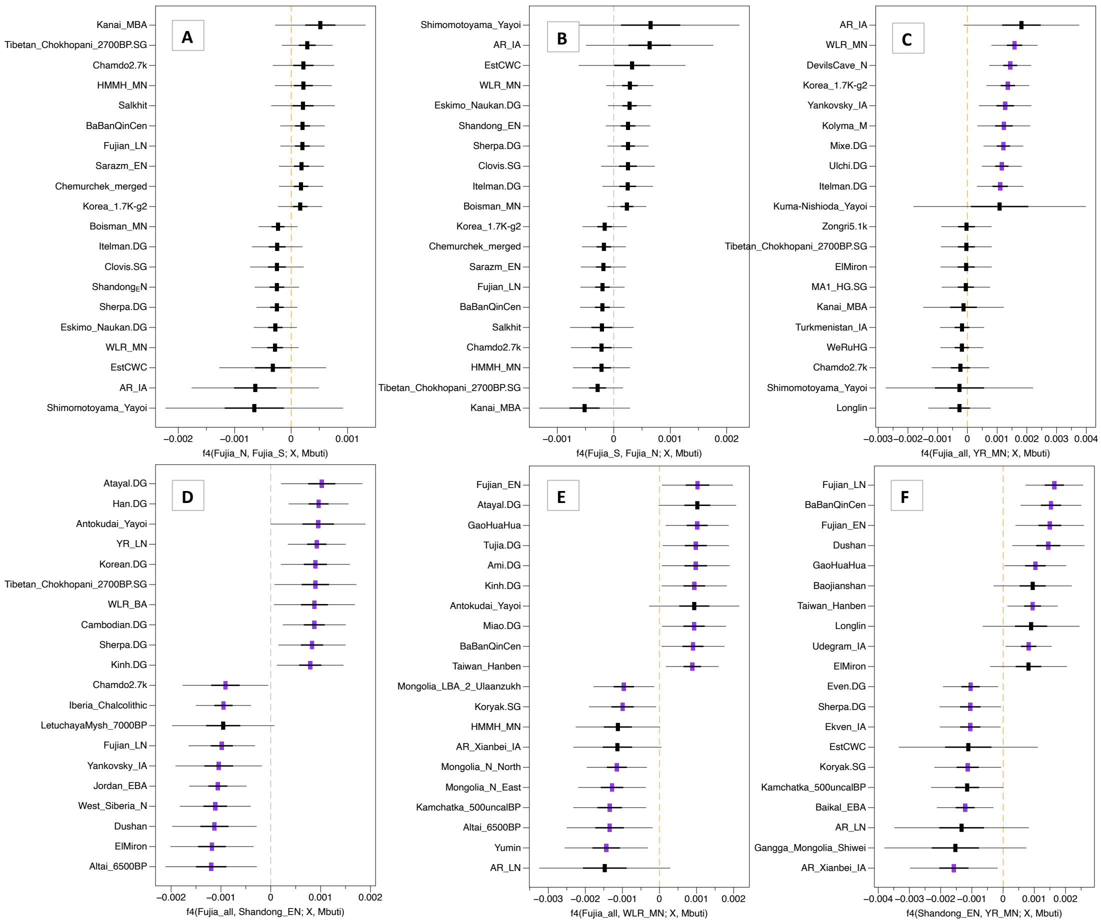

Figure S8. Symmetry tests between Fujia and other relevant ancient population from Shandong and nearby regions. We present 10 most positive (upper side) and 10 most negative (lower side) *f_4*-statistics across 334 world-wide populations. Horizontal bars represent the point estimate ± 3 (thin) and ± 1 (thick) s.e.m, respectivley. s.e.m. are estimated using 5 cM block jackknifing. *F_4* statistics deviating three s.e.m. or more from zero are marked in purple.

Supplementary Note 4. Biological relatedness of Fujia individuals

Kinship analysis

We employed three distinct methods to ascertain the biological relatedness among the Fujia site individuals: READ[17], KIN[18] and ancIBD[19]. These approaches were carefully selected to ensure robust analysis from different analytical angles. Our findings were consistent across all methods, affirming the reliability of the observed kinship patterns within the population. This consistency not only underscores the accuracy of each method but also provides a comprehensive view of the genetic relationships at the Fujia archaeological site, enhancing our understanding of the social and familial structures of this ancient community.

At the Fujia archaeological site, our study identified five pairs of first-degree relatives (Figure S9). N12 and N13 are likely sisters; S22 (male) and S33 (female) are siblings; and S03 and S19 form a mother-daughter pair, both identified as females. Furthermore, S03 is classified as a second-degree relative to both S22 and S33, supported by a KIN with a log likelihood ratio exceeding 6. S19 is a third-degree relative to both S22 and S33, reinforcing S03's role as S19's mother. This kinship configuration is illustrated in the pedigree tree shown in Figure S9. Additionally, S41 (female) and S11 (male), along with S52 (female) and S35 (male), are identified as parent and child pairs. The consistent mitochondrial haplotypes across the Fujia site, following a maternal inheritance pattern, make it challenging to discern whether these parent-child pairs are mother-son or father-daughter. No significant first- or second-degree interrelations were observed among other individuals at the site.

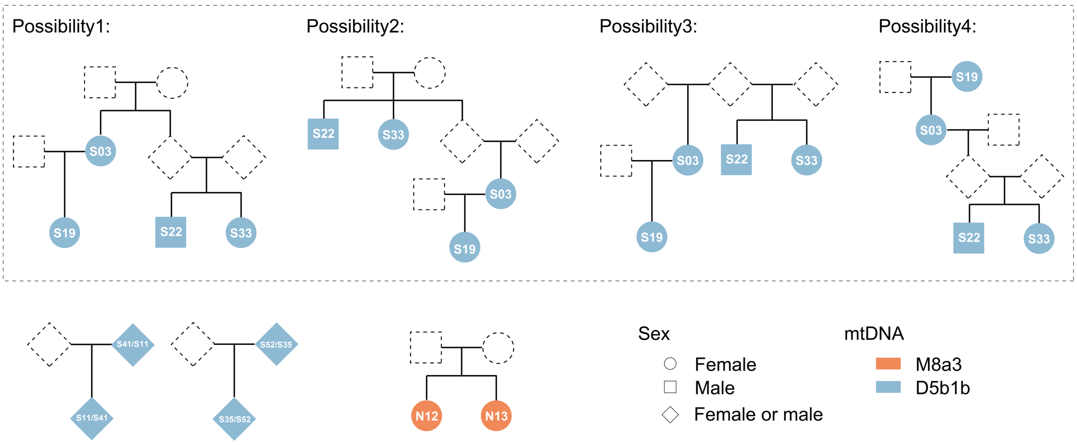

Figure S9. Family tree of Fujia individuals. The phylogenetic structure was established by both genetic relatedness estimated from autosomal data and uniparental genetic data from both mtDNA and Y chromosome.

Regarding ancIBD results, we compiled summary statistics for each pair where IBD was detected, noting shared IBD segments over 12 cM (Extended Figure 2C), 16 cM (Figure S10A), and 20 cM (Figure S10B). The length of shared IBD provides insights into the biological relatedness of the paired individuals. We also used the sum of IBD lengths to validate previously identified kinship relatedness. The IBD results align well with the estimation from KIN, where parent-child pairs share about 3,200 cM of IBD,

and siblings share approximately 2,500 cM (Extended Figure 2A and 3C). Additionally, within either the Fujia_S or Fujia_N groups, numerous second- and third-degree affinities were observed among individuals per cemetery. We also noted several third- to sixth-degree relationships between individuals from the two cemeteries, suggesting intermarriage between these groups, but buried back to their maternal ancestor's cemetery. This complex network of relationships highlights significant social and genetic interactions within the Fujia community.

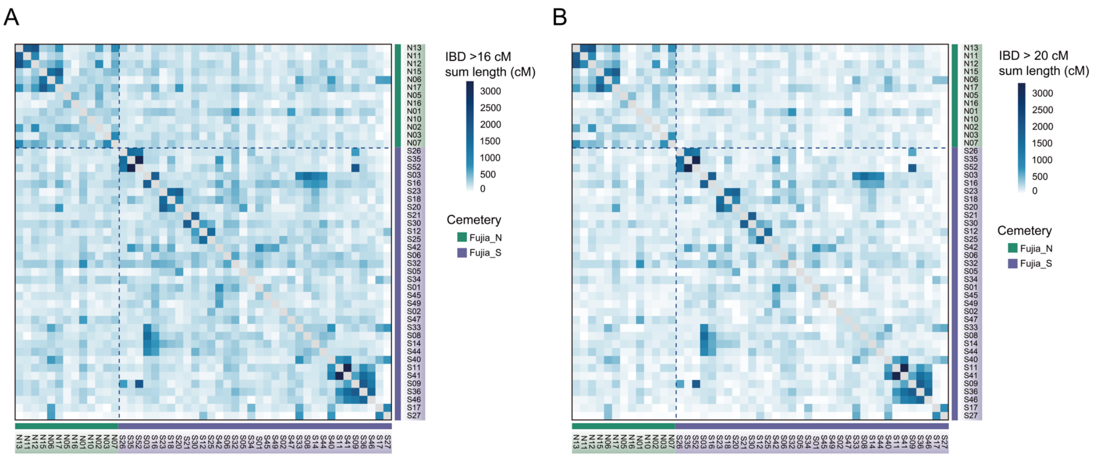

Figure S10. Heatmap of shared IBD (>16 cM) of Fujia individuals (n=44). **B.** Heatmap of shared IBD (>20 cM) of Fujia individuals (n=44).

Spatial distribution of paired individuals

The spatial distance between paired individuals interred within the same cemetery was quantified to explore potential correlations with genetic relatedness, using parameters such as pairwise mismatch rate (PMR), individual-f3 statistics, and shared identical-by-descent (IBD) segment lengths. Our analysis, as detailed in Extended Figure 2A, 2B and 2C; Figure S11A and 11B, revealed no significant correlation between the spatial proximity of burial sites and genetic relatedness. This suggests that closely related individuals were not necessarily buried nearby each other, indicating that the placement of individuals within the cemeteries might have been influenced by other factors other than familial relationships.

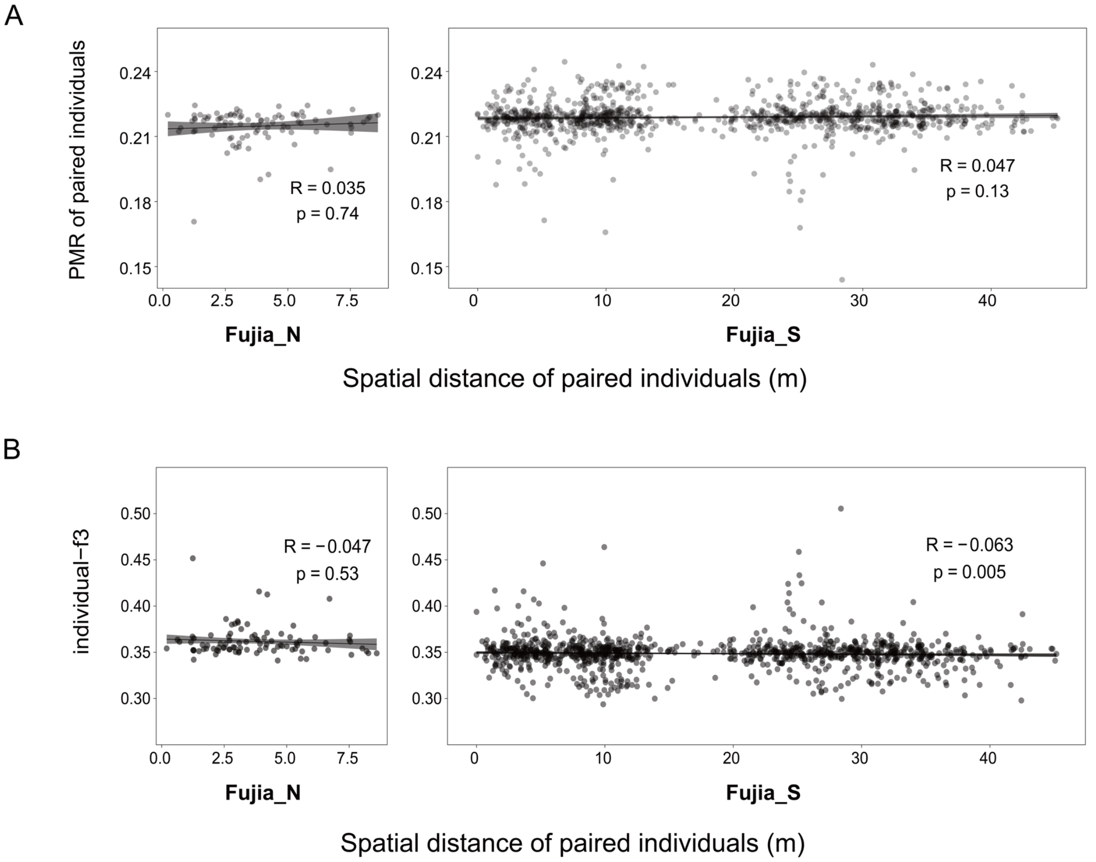

Figure S11. A. Two-sided spearman correlation of burial spatial distance and genetic affinity (PMR) among paird individuals. **B.** Two-sided spearman correlation of burial spatial distance and genetic affinity (individual-f3) among paird individuals. The coefficients and p-values were indicated on the plot. The coefficients and p-values were indicated on the plot.

Runs of Homozygosity

We utilized the hapROH software (version 0.64)[20] to analyze runs of homozygosity (ROH) in 47 individuals (13 from Fujia_N and 34 from Fujia_S) who exhibited more than 200,000 SNPs overlapping with the '1240k' dataset. Using the 1000 Genome Project haplotypes as the reference panel, we adhered to the default parameters set by the software. For each individual, we calculated a variety of ROH summary statistics, including the number and cumulative length of ROH segments exceeding thresholds of 4, 8, 12, and 20 cM, as well as the maximum length of ROH observed. Our findings indicated that ROH was present in all 47 individuals from the Fujia site, as shown in Figure S12A and 12B. We also analyzed the distribution of ROH lengths. Both Fujia_N and Fujia_S exhibited similar patterns, where short ROH segments (less than 8 cM) were predominantly observed, deviating from the distribution expected for offspring from closely related unions, as illustrated in Figure S12A and 12B. The high prevalence of short ROH segments (4–8 cM), accounting for 27–56% and 14.6%–83.9% of the total ROH length in Fujia_N and Fujia_S individuals respectively, suggests background parental relatedness due to a limited mating pool. This pattern reflects the genetic

structure within the community and provides insights into the demographic history of the populations at the Fujia site.

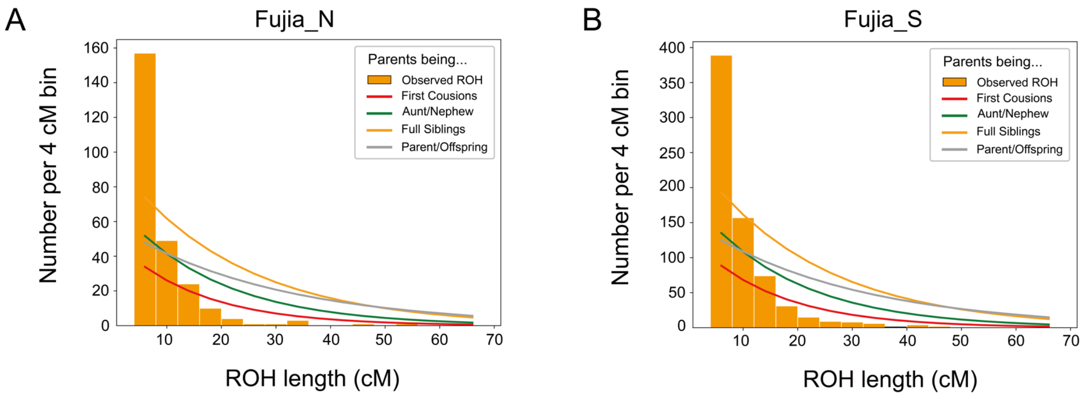

Figure S12. ROH patterns of Fujia. Combined histogram of ROH length of Fujia_N (A) (n=13) and Fujia_S (B) (n=34). Both cemeteries show similar pattern which short (<10 cM) ROH with high abundance and ROH does not fit well with expected curve for certain parental relationships.

Supplementary Note 5. Stable isotope data analysis

Strontium isotope analysis

Regional bioavailable strontium isotope baseline

We conducted high-resolution sampling of modern plants near the Fujia site based on a 1:200,000 geological map[21]. Geologically, the Fujia site is situated at the intersection of the alluvial plain of the northern foothills of Lushan Mountain and the Yellow River floodplain. The region contains diverse Quaternary deposits, including alluvial plain deposits (Ql in Figure S13), extensive coastal Quaternary deposits (Qw), and loess deposits from the foothill and valley areas (Qd, Qsh, and Qy). Despite all being of Quaternary age, these diverse deposit types across the study area likely produce distinct strontium isotope signatures. South of the site is Lushan Mountain, which reaches an elevation of approximately 500 meters. This area is dominated by Cambrian, Ordovician, and Carboniferous rocks—primarily limestone, dolostone, and shale—along with Quaternary deposits.

Our results indicate that $^{87}Sr/^{86}Sr$ ratios of plants differ significantly among these lithologies. For example, plants growing in the Quaternary alluvial plain deposits (Ql) near the Fujia site display an average $^{87}Sr/^{86}Sr$ ratio of 0.711222 ± 0.000305 (n = 22), which is substantially higher than that observed in plants from Quaternary deposits (Qd, Qsh, and Qy) in the foothill and valleys of the Lushan Mountain (0.710588 ± 0.000177, n = 6). Coastal Quaternary deposits (Qw), influenced by marine Sr sources such as seawater and seawater aerosols[22], yield lower $^{87}Sr/^{86}Sr$ values (0.710676 ± 0.000443, n = 4), although one sample collected further inland exhibits a higher $^{87}Sr/^{86}Sr$ ratio exceeding 0.711. Additionally, Carboniferous limestone from Lushan Mountain exhibits relatively low values (0.710457 ± 0.000122, n = 2), consistent with the generally low strontium isotope values (~0.708) reported in carbonate rocks[23], although these can rise due to later metamorphic processes[24]. In contrast, Permian terrigenous clastic rock (e.g. sandstone and mudstone) show higher $^{87}Sr/^{86}Sr$ ratios (0.711582 ± 0.00015, n = 3), likely reflecting the influence of older granitic or rubidium/potassium-rich parent rocks.

Notably, Ordovician dolostone and limestone (0.711075 ± 0.000204, n = 10) partially overlap in $^{87}Sr/^{86}Sr$ values with the alluvial plain deposits at the Fujia site. This overlap unnderscores a common challenge in strontium isotope sourcing, where baseline $^{87}Sr/^{86}Sr$ values may be similar across regions with both comparable and distinct geologic settings. Therefore, evaluating the migration patterns of individuals from Fujia requires corroboration from multiple lines of evidence.

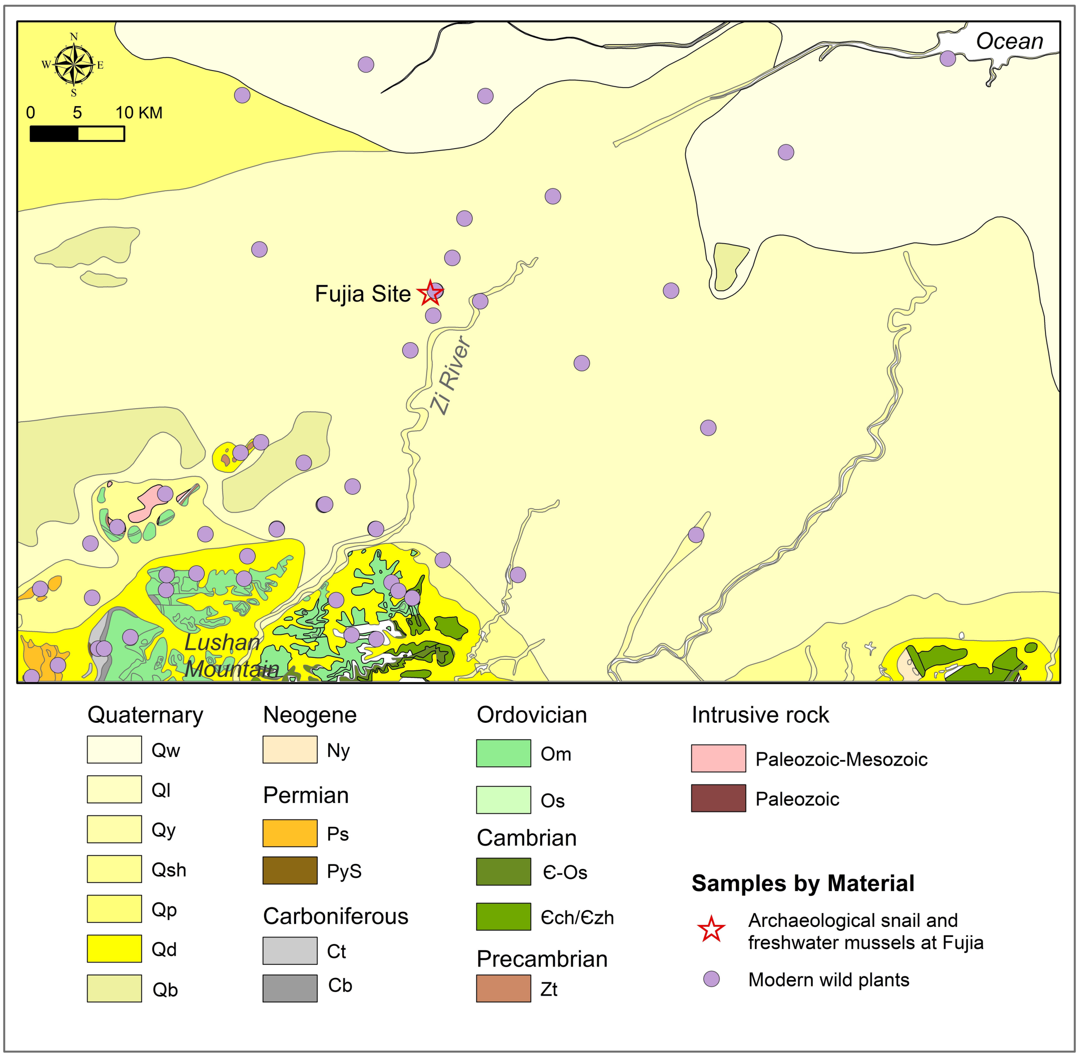

Figure S13. Geological map of the study area and sample locations mentioned in the text. Qw (Huaibei formation), gray siltstone and mudstone mainly distributed along the coast; Ql (Linyi formation), piedmont alluvial plain mainly composed of loose yellow sand, silt, and silty clay; Qy (Yihe formation), fluvial sediments, including sand, gravel, and silt; Qsh (Shanqian formation), residual deposits consisting of yellow-brown and reddish-brown sandy clay; Qp (Pingyuan formation), brown-yellow silty clay; Qd (Dazhan formation), yellow silt, sandy clay, and gravel layers; Qb (Baiyunhu formation), lacustrine and swamp sediments, mainly consisting of black and dark brown silty clay and clay; Ny (Yaoshan formation), yellow silt, sandy clay, and gravel layers; Ps (Shihezi formation), thick coarse quartz sandstone, yellow-green feldspathic quartz sandstone, and fine sandstone; PyS (Shanxi formation), gray to dark gray mudstone and silty mudstone; Ct (Taiyuan formation), gray to dark-gray limestone, marl, mudstone, and shale interbedded with several layers of sandstone; Cb (Benxi formation), limestone, marl, mudstone, feldspathic quartz sandstone, and shale; O_m (Majiagou formation), mud limestone and microcrystalline dolostone; Os (upper Sanshanzi formation), gray-yellow and rose-red flint nodules in fine-grained dolostone; Є-Os (lower Sanshanzi formation), medium to thick layers of dolostone; Єch/Єzh (Chaomidian formation/Zhangxia formation), yellow-brown shale, thick limestone; Zt

(Tumen group), light gray microcrystalline dolostone, gray-yellow fine sandstone, and siltstone. The basemap was plotted from an open source data of 1:200,000 geological map [21](https://geocloud.cgs.gov.cn/common-search/search/detail?globalId=cpgl_dzcp_43166dd81dfd430e87755cb2f36ceee7&networkType=extranet&table_name=cpgl_dzcp&isAccurate=false&keyword=J-50-29) by ArcGIS.

Fujia individual migration patterns

From a large-scale perspective, we first compare the $^{87}Sr/^{86}Sr$ ratios of Fujia individuals with those of plants from different geological units across the study area. The $^{87}Sr/^{86}Sr$ ratios of all Fujia individuals (ranging from 0.711030 to 0.711554) fall within the range of plant $^{87}Sr/^{86}Sr$ ratios from the piedmont Quaternary alluvial plain deposits (Ql) where the Fujia site is located, with some individuals also overlapping with the ranges of Ordovician dolostone and limestone in Lushan Mountain (Figure 4 in the main text). This clearly distinguishes the Fujia individuals from those associated with other geological units in the region.

To further constrain their origins, we incorporate oxygen isotope analysis, a widely used method for tracing ancient migration and mobility (reviewed in Pederzani and Britton 2019[25]). Pevious studies demonstrated that the $\delta^{18}O$ values of the 23 individuals excavated from the Fujia site are remarkably clustered (with 1 SD < 1‰)[26], exhibiting a very minimal range even for a single population. Typically, $\delta^{18}O$ variation is observed even within populations from the same area due to factors such as differences in water sources, cooking methods, and individual physiological conditions[25]. Notably, elevation effects and water source differences would typically result in greater $\delta^{18}O$ variation within a population. Thus, we infer that all Fujia individuals likely lived within the Quaternary alluvial plain deposits surrounding the site, sharing a common water source, rather than originating than from the high-elevation Ordovician rock formations in Lushan Mountain.

Even within the Quaternary alluvial plain deposits, some variation in $^{87}Sr/^{86}Sr$ ratios is observed. Therefore, we also discuss it from a more local-scale perspective. Our results show that the $^{87}Sr/^{86}Sr$ values of all Fujia individuals fall within the $^{87}Sr/^{86}Sr$ range of plant samples within a 10 km radius of the Fujia site (~0.7107–0.71153). Additionaly, a snail sample excavated from the Fujia site has a $^{87}Sr/^{86}Sr$ ratio of 0.711383, and two freshwater mussel samples yield $^{87}Sr/^{86}Sr$ ratios of 0.711393 ± 0.000173. These values are consistent with both the local plant values and the majority of the $^{87}Sr/^{86}Sr$ ratios of Fujia individuals. Future analyses of additional low-mobility faunal remains, if available, would further validate this conclusion.

Furthermore, in the absence of animal samples or external Sr sources (such as marine Sr, as discussed in this study and in Wright 2005[27]), Wright's method of normal distribution testing was applied to identify and exclude outlier $^{87}Sr/^{86}Sr$ values. Wright suggested that after removing outliers, the remaining values should fit a normal distribution, which can be then used to estimate the local $^{87}Sr/^{86}Sr$ range. A Shapiro–Wilk test confirmed that the strontium isotope data from the Fujia individuals conform to a normal distribution (p-value > 0.05), with no outliers detected and no further data

trimming required. This supports the hypothesis that the Fujia individuals were likely a local population.

Ancient DNA (aDNA) analyses further corroborate the localization of Fujia individuals. First, individuals from both the Fujia_S and Fujia_N groups show the highest genetic drift with each other compared to other nearby populations, as seen in Figure S11, and form a tight cluster in principal component analysis (PCA) in Figure S6. This suggests that the Fujia individuals are closely related to each other and were not subject to long-distance migration or recent gene flow from outlier individuals. Second, the exceptionally high runs of homozygosity (ROH) observed in Fujia individuals (Figure S12), which are absent in other Shandong populations and more broadly worldwide, indicate high levels of local endogamy. Considering the long duration of cemetery use (~250 years, approximately 10 generations), this observation strongly suggests that the Fujia individuals were localized.

Based on multiple lines of evidence, including regional and local plant and microfauna strontium isotopes, oxygen isotopes, normal distribution test, and aDNA, we conclude that the individuals excavated from the Fujia archaeological site were local inhabitants.

Accessing marine Sr influence

Paleoclimate reconstructions suggest that the Fujia site was likely located near the coastline during the mid-Holocene[28], within a few kilometers of the shoreline (Figure 1B). Consequently, it is important to assess the potential impact of marine food consumption on the $^{87}Sr/^{86}Sr$ signatures of the Fujia individuals. Typically, individuals consuming substantial amounts of marine products or sea salt would exhibit $^{87}Sr/^{86}Sr$ ratios close to marine $^{87}Sr/^{86}Sr$ values (~0.709)[29]. However, our results show that the $^{87}Sr/^{86}Sr$ ratios of all Fujia individuals are similar to those of local plants, snail shell, and freshwater mussels, and are much higher than marine $^{87}Sr/^{86}Sr$ values. This suggests that the Fujia individuals likely did not heavily rely on marine resources, though some level of consumption cannot be entirely ruled out.

At present, we are unable to precisely estimate the amount of marine products consumed by the Fujia individuals. Unlike carbon and nitrogen isotopes, which are used to infer dietary components, strontium isotopes behave differently and do not allow for direct inference of Sr isotope compositions from dietary isotopes. Strontium intake is not solely derived from food; it also comes from water sources, salts, and other environmental factors[30–32]. Additionally, strontium concentrations vary across plant, marine, and terrestrial animal food sources, complicating the quantification of marine product consumption through Sr isotopes. Accurate estimation would require detailed knowledge of the Sr isotope and concentrations in different food types and water sources, as well as measurements of the Sr concentration and isotopic values in individual samples.

Limitations of using modern plants for strontium isotope baselines

A key limitation of this study is the reliance on modern plant samples to construct a high-resolution bioavailable strontium isoscape for the area surrounding the Fujia site. Although modern plants have been widely used as a reliable proxy for local $^{87}Sr/^{86}Sr$

values in numerous isoscape and mobility studies[33,34], the extent to which these samples are affected by anthropogenic contamination is rarely evaluated and remains inherently difficult to assess—particularly in regions with intensive agricultural and industrial activity, such as our study area.

In contrast, low-mobility archaeological faunal remains (e.g., rodent tooth enamel) are less susceptible to modern contamination and may provide a more accurate reflection of prehistoric local strontium isotope baselines. However, such samples are currently underrepresented in both global and Chinese $^{87}Sr/^{86}Sr$ isoscape datasets[35,36], as they are often overlooked during archaeological excavation and sampling. We therefore strongly encourage future research to prioritize the systematic collection and analysis of low-mobility faunal remains from Fujia and surrounding archaeological sites—ideally at the regional or even national scale. These samples are essential for building more robust and spatially refined strontium isotope baselines, which are critical for high-resolution reconstructions of past human mobility and migration in this region.

Carbon and nitrogen isotope analysis

Comparisons with other Middle/Late Dawenkou archaeological sites

This study integrates previously published carbon and nitrogen isotope data from both human and animal remains in the Dawenkou culture region, offering a more comprehensive understanding of dietary practices during this period. Dong *et al.* 2021[4] conducted an in-depth comparison of Middle and Late Dawenkou archaeological sites, providing valuable insights into regional dietary patterns. Their findings indicate that northern Dawenkou sites, such as Fujia, Dongjiaying, Beiqian, and Jiaojia, exhibit higher $\delta^{13}C$ values, which they attribute to a diet rich in millets and C_4-feed pigs. In contrast, individuals from southern sites such as Xigongqiao, the Dawenkou type site, and Liangwangcheng exhibit a wide range of $\delta^{13}C$ values, suggesting that some individuals ($\delta^{13}C$ values between –18‰ and –12‰) consumed a mixed C_3- and C_4-based diet, while others ($\delta^{13}C$ values above –12‰) primarily consumed C_4-based foods. This is likely due to a greater reliance on millet, rice, and deer in the south, where rice and millet could have been cultivated together during this period[4]. Furthermore, nitrogen isotope and zooarchaeological evidence suggest that coastal sites (e.g. Fujia and Wucun) may have incorporated marine resources, and many riverside sites, including Fujia near the Zi River, also likely consumed freshwater aquatic resources. However, distinguishing between terrestrial and aquatic animal protein consumption remains challenging, as the bulk isotopic methods do not yet provide a clear differentiation between these sources.

Pigs and deer are the most abundant mammals across these sites. Wild herbivores (i.e., deer) from Beiqian, Liangwangcheng, and the Dawenkou type site all show C_3 diets based on the consumption of plant protein, with average $\delta^{15}N$ values of 3.1‰, 4.1‰, and 2.2‰, respectively (Supplementary Data S2). These values align with the local C_3 environmental background and are typical for herbivores. However, pig isotope values vary considerably between sites. Pigs from Dongjiaying and Fujia exhibit a typical C_4 diet, similar to that of humans at these sites, while pigs from the Dawenkou type site show C_3-based diets, akin to those of deer. Pigs from Beiqian and Liangwangcheng

display a wide range of $\delta^{13}C$ variation. In terms of nitrogen isotopes, pigs from Fujia have the highest mean $\delta^{15}N$ value (7.0‰) with the lowest standard deviation (0.6‰). In contrast, pigs from the Dawenkou type site exhibit the lowest $\delta^{15}N$ values (3.7‰), likely indicative of a C_3 plant-based diet. The variations in pig isotope values between sites likely reflect differences in animal husbandry practices across different Dawenkou sites or the presence of wild boar. Nevertheless, pigs from Fujia exhibit remarkable dietary consistency, suggesting the use of similar C_4-based food resources in pig husbandry across the site.

The higher $\delta^{15}N$ values of Fujia individuals compared to other Dawenkou sites (e.g. Beiqian; Fig. 4B and S14) may be influced by local environmental factors or dietary practice. Fujia was located ~5.8 km from the coastline during the Late Dawenkou period, based on mid-Holocene sea-level reconstructions along the southern Bohai Bay (Fig. 1B). Proximity to the coast likely contributed to saline soil conditions, potentially elevate baseline $\delta^{15}N$ values in local ecosystems[4,37], and consequently can increase the $\delta^{15}N$ values of pigs and humans at Fujia. Additionally, the consumption of marine and/or freshwater resources may have contributed to these elevated $\delta^{15}N$ values, as evidenced by the presence of marine mollusks at nearby sites (e.g., Wucun) and the widespread discovery of freshwater mussels at Fujia (located near the Zi River) and other Dawenkou sites (summarized in the supplementary materials of Dong *et al.* 2021[4]). Future studies should incorporate compound-specific stable isotope analysis on human samples, as well as plants and wild animals from the site and surrounding areas, will be crucial for more precisely distinguish the relative contributions of environmental and dietary factors.

Unlike previous study, we expand the scope of analysis by measuring the isotopic breadth of foods consumed by populations during the Dawenkou period using Stable Isotope Bayesian Ellipses (SIBER) metrics[38]. This approach enables us to examine intra-regional dietary differentiation across distinct archaeological sites, providing a more nuanced understanding of the dietary niches occupied by these communities.

Our results indicate significant differences in total areas (TA), SEA_B sizes, and SEA_C core niches between the sites (Figure S14). Specifically, the Fujia population exhibits both high $\delta^{13}C$ and $\delta^{15}N$ values, indicating a highly consistent diet. The Fujia population occupies the smallest dietary niches, with a SEA_C core niche area of just 0.87‰^{2}, suggesting a homogeneous food consumption pattern. In contrast, individuals from the Xigongqiao site occupy the largest dietary niche area (10.05‰^{2}of SEA_C core niches), with a significantly broader isotopic range for both carbon and nitrogen. This indicates that the Xigongqiao population had a more diverse dietary strategy, encompassing a wider spectrum of animal protein consumption (from low to high) and a mix of C_3- and C_4-based diets, in contrast to the more specialized dietary pattern observed at Fujia.

Other Dawenkou sites, such as Jiaojia (2.48‰^{2}), Beiqian (2.45‰^{2}), and Dawenkou (2.12‰^{2}), exhibit relatively narrow dietary niches (Figure S14), indicating that these populations also maintained more uniform dietary patterns, though with some variability. Notably, Dongjiaying (4.68‰^{2}) and Liangwangcheng (3.60‰^{2}) show the second-largest dietary niches. Dongjiaying's isotopic niche breadth primarily reflects a wider $\delta^{15}N$ range, likely linked to differing consumption of protein sources within the

population. The broader dietary niche in Liangwangcheng can be attributed to variations in carbon isotopes, with some individuals consuming a mixed C_3- and C_4-based diet, while others had a predominantly C_4-based diet.

The pronounced differences in the dietary niches of the Fujia population suggest that the Fujia society may have had a more consistent dietary structure, potentially indicative of a more closely-knit or less socially stratified community. This is consistent with the lack of evidence for differentiation in mortuary treatment among individuals at Fujia. In contrast, dietary practices at other sites, such as Xigongqiao and Liangwangcheng, display greater diversity, which correlates with some degree of differentiation in mortuary treatment, as reflected in the varying number of grave goods (summarized in the supplementary materials of Dong *et al.* 2021[4]). Specifically, Chen *et al*. 2019[39] reported that individuals from elaborate burials at late Dawenkou sites had significantly higher nitrogen isotope values compared to those from simpler burials, suggesting greater access to preferred foods, such as animal proteins with elevated $\delta^{15}N$ value.

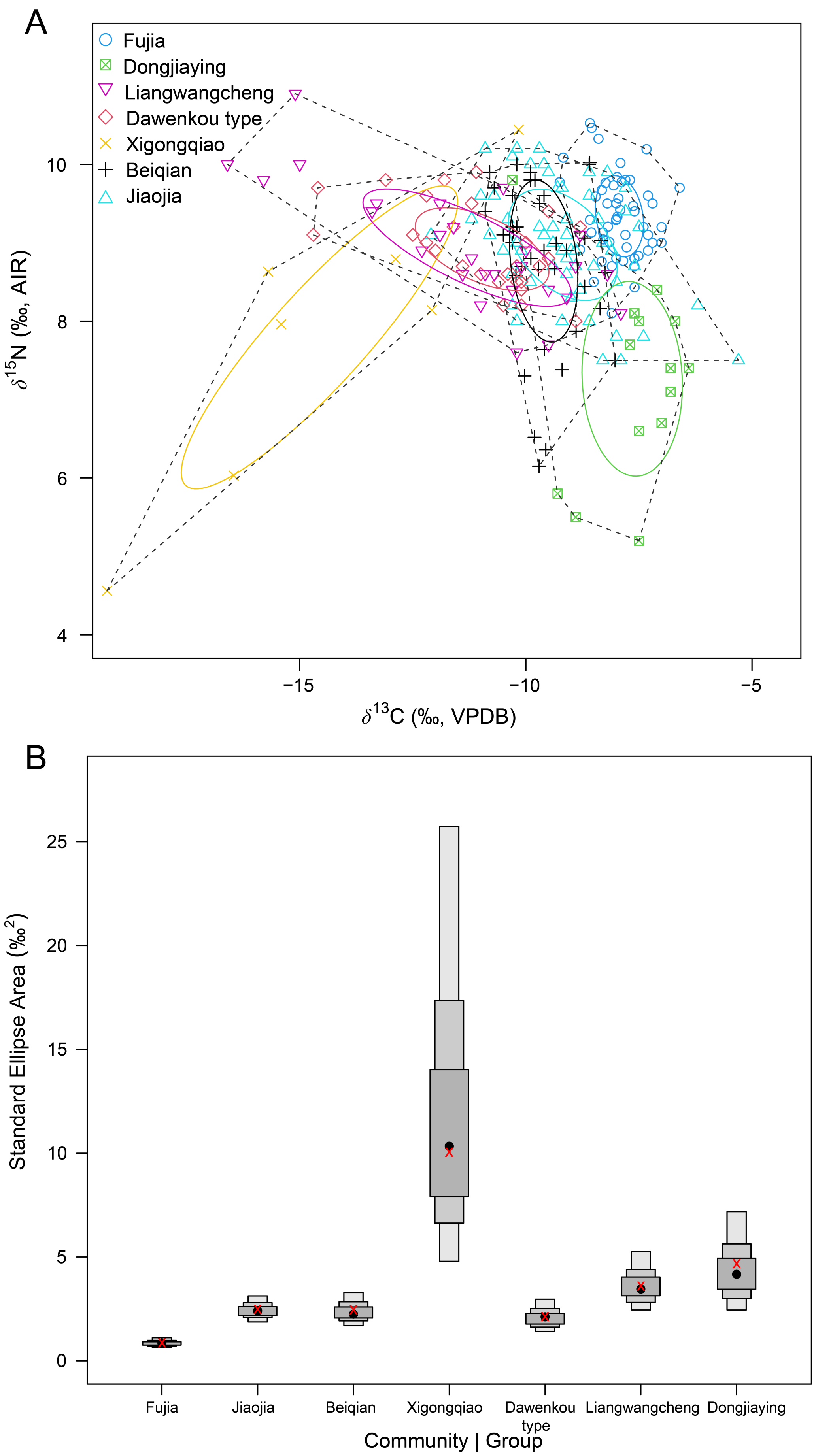
A
Fujia
Dongjiaying
Liangwangcheng
Dawenkou type
Xigongqiao
Beiqian
Jiaojia
δ15N (‰, AIR)
δ13C (‰, VPDB)
B
Standard Ellipse Area (‰2)
Fujia
Jiaojia
Beiqian
Xigongqiao
Dawenkou type
Liangwangcheng
Dongjiaying
Community | Group

573

Figure S14. Carbon and nitrogen isotope analysis. A. Convex hull area (TA, indicated by dashed lines) and Standard Ellipse Area corrected for small samples (SEA_C) for human samples from Dawenkou archaeological sites using the Stable Isotope Bayesian Ellipses (SIBER) package in R version 4.3.2. SEA_C relied on the spread of bivariate stable isotope data are compared using this method. Specifically, SEA_C provides information about the core aspects of a population's niche (explaining 40% of the information) and is less sensitive to outliers and small samples ($n < 30$)[40]. B. Bayesian Standard Ellipse Area (SEA_B) for human isotope data from the seven archeological sites analyzed. Shaded boxes represent the 50%, 75%, and 95% intervals of the Bayesian posterior distribution. Red crosses represent SEA_C. Sample sizes in Figures A and B are as follows: Fujia (n=51), Jiaojia (n=60), Beiqian (n=38), Liangwangcheng (n=27), Dawenkou type (n=29), Xigongqiao (n=7), and Dongjiaying (n=14). Published isotope data can be found in Supplementary Data S2.

Sex Differences in Diet

Examining sex-related dietary differences is crucial for understanding the social structure and subsistence strategies of ancient populations. We integrated available carbon and nitrogen isotope data from sex-identifiable individuals across multiple Middle/Late Dawenkou culture sites (Supplementary Data S2). While previous studies have suggested that some Middle to Late Dawenkou sites may have exhibited social divisions of labor and varying social statuses between males and females, our *t*-test analysis reveals no statistically significant differences (p-value > 0.05) in either carbon or nitrogen isotope ratios by sex at any site (Figure S15). This finding suggests that, on a broader scale, dietary patterns for males and females within the Dawenkou culture were largely consistent.

Despite the absence of significant isotopic differences overall, subtle variations are still apparent (Figure S15). For example, at Liangwangcheng, female individuals exhibited greater $\delta^{13}C$ variation than males, driven primarily by four adult females who appeared to consume more preferred foods, including higher amounts of rice, meat, and/or aquatic resources[41]. At the Dawenkou type site, female $\delta^{13}C$ values were slightly higher (by approximately 1‰) than those of males, possibly indicating marginally greater millet consumption[39]. However, $\delta^{15}N$ values sugget that both sexes had similar protein intakes[39]. At Beiqian, females showed more variability in both carbon and nitrogen isotopes than males, potentially reflecting diverse dietary practices among women at this site (Figure S15). In contrast, at Fujia, there were no significant differences in either mean isotope values or their standard deviations between males and females, suggesting that dietary practices at Fujia may have been more uniform across sex.

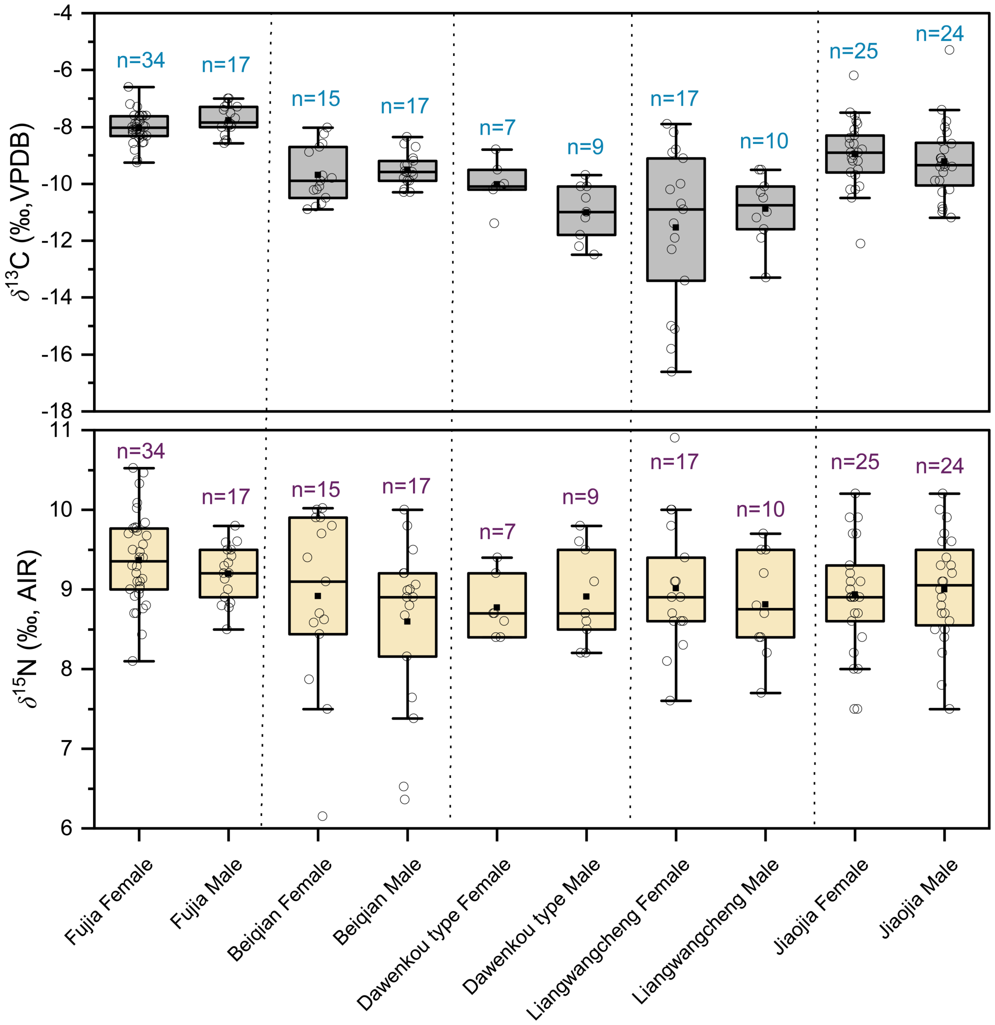

Figure S15. Comparison of carbon and nitrogen isotopes between males and females from various Dawenkou period sites. Published isotope data can be found in Supplementary Data S2. No statistically significant differences (p-value > 0.05) were observed in carbon or nitrogen isotope ratios between sexes at any site by two-sided *t*-test. Sample sizes were labelled on the top of the boxplots. Boxplots display the median (centre line), first and third quartiles (lower/upper hinges), and whiskers extending up to 1.5× IQR from the hinges.

Supplementary Note 6. Grave value of the Dawenkou cultural archaeological sites

Grave Value (GV) is an indicator in burial study in archaeology specifically designed to evaluate the rank of each grave within a cemetery by quantitative method. It is assumed that the value of a grave is determined by the different type of items buried in the grave, including the funerary objects, utensils and others, in terms of their frequency of occurrence across all graves in the cemetery. Items exclusively associated with large graves are considered belonging to those who hold higher social status[42]. In practice, GV can be calculated by first categorizing and then calculating the occurrence frequency of each type of items across the graves in a cemetery using the following formula provided by Jorgenson (1987)[43].

Type Value (TV) = Total Amount of Graves / Frequency of Type
GV = Sum of all Type Values

Flad (2001) improved this method in his study of a Bronze Age cemetery, Dadianzi, in northern China[44]. Besides making a differentiation between 'Ceremony Grave Value' and 'Deceased Grave Value', he optimizes the calculation of GV based on the different attributes of grave types. Prior to statistical analysis, qualitative analysis is firstly used to decide whether to consider the number of grave goods or only their presence. For prestige good, the mere presence confirms the value of the grave, while for more commonly funerary objects, both the presence and quantity are considered, such as pottery and jade. For quantity, *natural breaks* method is applied to assign different values to the numbers of items. For instance, in the Dadianzi cemetery with 742 graves, jade was found in 203 graves. Using *natural breaks*, they can be categorized into three levels: 6 graves contained 84-859 jades, 11 graves contained 9-52 jades, and 186 graves contained 1-7 jades. Their TVs are then calculated as 123.67 (742/6), 43.65 (742/(6+11)), and 3.66 (742/(6+11+186)).

This study follows Flad's method in differentiating the Dawenkou grave values into two types: the presence and the quantity. The 'presence items' include 1) wooden coffins, 2) stones, bones, or horn artifacts, and 3) carved cylinders, as special artifacts, are typically made of ivory or bone and coexist with axes or deer antlers, which are fixed on the heads of those tool handles. The potteries and jades are the 'quantity items', with their TVs calculated according to Flad's *natural breaks* method. At last, the sum of both presence and quantity values yields the overall grave values. Instead of evaluating GVs within individual cemetery, all Dawenkou cemeteries involved in this study are calculated together in order to make comparations between sites, which includes: Xixiahou, Qianbuxia, Liulijing, Jianxin, Shangzhuang, Sanlihe, Xigongqiao, Dazhujia, Dawenkou, Yedian, Jiaojia, Gangshang and Fujia (Supplementary Data S7).

In total, there are 1067 graves were included in 13 Dawenkou cemeteries. For the 'presence item', 138 graves contain coffins with TV of 7.73 (1067/138), 378 graves with stone, bone, or horn artifacts, with TV of 2.82 (1067/378). 26 graves contain bone- or ivory-carved cylinders, with TV of 41.04 (1067/26). The jade (including turquoises) TV are calculated by *natural breaks* to be categorized into three levels: 118.56 (1067/9) with more than 5 jades, 35.57 (1067/(9+21)) with 2-4 jades and 14.23(1067/(9+21+45))

with only one jade. The pottery is categorized into five levels: 59.28 (1067/18) with 58 or more potteries, 20.92(1067/(18+33)) with 34-57 potteries, 8.89 (1067/(18+33+69)) with 16-33 potteries, 2.95 (1067/(18+33+69+242)) with 5-15 potteries, and 1.48 (1067/(18+33+69+242+361)) with 1-5 potteries. Then the total Grave Value = Coffin TV + Stone, Bone, or Horn artifacts TV + Carved Cylinder TV+ Jade TV + Pottery TV.

A battleship plot (Figure S16) based on the percentage of four levels of GV (0, 0~10, 10~50, 50~300) for each cemetery demonstrates the different patterns of social stratifications on Dawenkou societies in Shandong. In contrast to the others, the graves at the Fujia site are predominantly concentrated in the lower value range, even more than 50% of graves with zero GV. This suggests less social wealth and stratification of Fujia in the overall Dawenkou society.

Besides the grave value, Fujia's pottery is comparatively crude, indicating lower levels of labor investments and technical proficiency[10]. Fujia graves exhibit reduced diversity and a lack of rare artifacts from long-distance exchanges or trade routes in burial assemblages compared to other Dawenkou sites[45]. This underscores the relative autonomy of Fujia and its limited engagement in the extensive social network characteristic of the broader Dawenkou Culture.

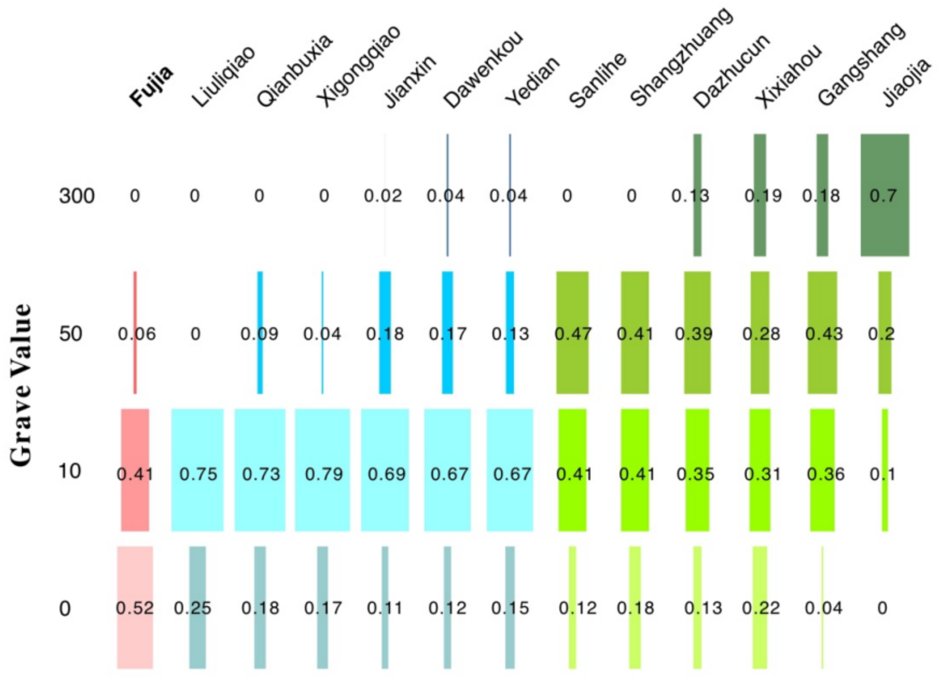

Figure S16. Grave Values of Dawenkou Cultural Cemeteries in Shandong province. The grave values for Dawenkou cemeteries are illustrated on the percentage within each site. They are generally classified into three patterns which are illustrated as red, blue and green colors. More than half tombs in Fujia have GV of zero. The cemeteries in Liuliqiao, Qianbuxia, Xigongqiao, Jianxin, Dawenkou and Yedian have most of GV between 0~10, indicating low social stratification. The cemeteries in Sanlihe, Shangzhuang, Dazhucun, Xixiahou, Gangshang and Jiaojia have relatively much higher

percentage of graves with higher GV, indicating more high-status person in the cemetery and higher social stratification. Due to the limit of publication of Jiaojia, the statistics on GV are most probably biased**.**

References:

1. Jin, G. New advances in the chronology of prehistoric archaeology in the Haidai region. *Wenbo China* (2023).
2. Wang, F. The Houli and Beixin Cultures. in *A Companion to Chinese Archaeology* 387–410 (John Wiley & Sons, Ltd, 2013). doi:10.1002/9781118325698.ch19.
3. Underhill, A. Dawenkou. in *Encyclopedia of Prehistory: Volume 3: East Asia and Oceania* (eds. Peregrine, P. N. & Ember, M.) 12–15 (Springer US, Boston, MA, 2001). doi:10.1007/978-1-4615-1189-2_2.
4. Dong, Y. *et al.* Social and environmental factors influencing dietary choices among Dawenkou culture sites, Late Neolithic China. *The Holocene* **31**, 271–284 (2021).
5. Dai, L. & Zhang, D. Pig management strategies during the early Dawenkou cultural period (ca. 6200–5600 BP): A stable isotopic analysis of animal remains from the Houtieying site, Anhui Province, China. *Quaternary International* **595**, 88–97 (2021).
6. Luan, F. The Dawenkou Culture in the Lower Yellow River and Huai River Basin Areas. in *A Companion to Chinese Archaeology* 411–434 (John Wiley & Sons, Ltd, 2013). doi:10.1002/9781118325698.ch20.
7. Manabe, Y. *et al.* Dental morphology of the Dawenkou Neolithic population in North China: implications for the origin and distribution of Sinodonty. *J Hum Evol* **45**, 369–380 (2003).
8. Lee, C. The Relationship between Intentional Dental Ablation and Hereditary Agenesis in Late Neolithic to Early Bronze Age China. in *A World View of Bioculturally Modified Teeth* (eds. Burnett, S. E. & Irish, J. D.) 0 (University Press of Florida, 2017). doi:10.5744/florida/9780813054834.003.0007.
9. Maisels, C. K. *Early Civilizations of the Old World: The Formative Histories of Egypt, The Levant, Mesopotamia, India and China*. (Routledge, London, 2001).
10. Li, Z., Wang J., Liu G. & Zhao Z. Excavation of the Fujia archaeological site in Guangrao County, Shandong. *Kaogu* 36-44+103+2 (2002).
11. Reimer, P. J. *et al.* The IntCal20 Northern Hemisphere Radiocarbon Age Calibration Curve (0–55 cal kBP). *Radiocarbon* **62**, 725–757 (2020).
12. Ramsey, C. B. Bayesian Analysis of Radiocarbon Dates. *Radiocarbon* **51**, 337–360 (2009).
13. Wang, T. *et al.* Human population history at the crossroads of East and Southeast Asia since 11,000 years ago. *Cell* **184**, 3829-3841.e21 (2021).
14. Yang, M. A. *et al.* Ancient DNA indicates human population shifts and admixture in northern and southern China. *Science* **369**, 282–288 (2020).
15. Ning, C. *et al.* Ancient genomes from northern China suggest links between subsistence changes and human migration. *Nature Communications* **11**, 2700 (2020).
16. Liu, L. *The Chinese Neolithic: Trajectories to Early States*. (Cambridge University Press, Cambridge, 2005). doi:10.1017/CBO9780511489624.
17. Kuhn, J. M. M., Jakobsson, M. & Günther, T. Estimating genetic kin relationships in prehistoric populations. *PLOS ONE* **13**, e0195491 (2018).
18. Popli, D., Peyrégne, S. & Peter, B. M. KIN: a method to infer relatedness from low-coverage ancient DNA. *Genome Biology* **24**, 10 (2023).

19. Ringbauer, H. *et al.* Accurate detection of identity-by-descent segments in human ancient DNA. *Nature Genetics* **56**, 143–151 (2024).
20. Ringbauer, H., Novembre, J. & Steinrücken, M. Parental relatedness through time revealed by runs of homozygosity in ancient DNA. *Nature Communications* **12**, 5425 (2021).
21. Li, C. National 1:200,000 Digital Geological Map (Public Version) Spatial Database. 90 GB Geoscientific Data and Discovery Publishing https://doi.org/10.23650/DATA.A.2019.NGA120157.K1.1.1.V1 (2019).
22. Evans, J. A., Montgomery, J., Wildman, G. & Boulton, N. Spatial variations in biosphere 87Sr/86Sr in Britain. *Journal of the Geological Society* **167**, 1–4 (2010).
23. Alexander Bentley, R. Strontium Isotopes from the Earth to the Archaeological Skeleton: A Review. *J Archaeol Method Theory* **13**, 135–187 (2006).
24. Zhang, J. *et al.* Water geochemistry of the rivers around the Taklimakan Desert (NW China): Crustal weathering and evaporation processes in arid land. *Chemical Geology* **119**, 225–237 (1995).
25. Pederzani, S. & Britton, K. Oxygen isotopes in bioarchaeology: Principles and applications, challenges and opportunities. *Earth-Science Reviews* **188**, 77–107 (2019).
26. Dong, Y. & Luan, F. Reflections on the Social Organization of the Late Dawenkou Culture: Evidence from DNA and Stable Isotopes. *Kaogu* **07**, 98–107 (2017).
27. Wright, L. E. Identifying immigrants to Tikal, Guatemala: Defining local variability in strontium isotope ratios of human tooth enamel. *Journal of Archaeological Science* **32**, 555–566 (2005).
28. Kong, Q. The fauna remains from Dawenkou culture site, Wucun, Guangrao county. *Haidai Kaogu* **1**, 122–123 (1989).
29. McArthur, J. M., Howarth, R. J. & Shields, G. A. Chapter 7 - Strontium Isotope Stratigraphy. in *The Geologic Time Scale* (eds. Gradstein, F. M., Ogg, J. G., Schmitz, M. D. & Ogg, G. M.) 127–144 (Elsevier, Boston, 2012). doi:10.1016/B978-0-444-59425-9.00007-X.
30. Lewis, J., Pike, A. W. G., Coath, C. D. & Evershed, R. P. Strontium concentration, radiogenic (87Sr/86Sr) and stable (δ88Sr) strontium isotope systematics in a controlled feeding study. *STAR: Science & Technology of Archaeological Research* (2017).
31. Fenner, J. N. & Wright, L. E. Revisiting the strontium contribution of sea salt in the human diet. *Journal of Archaeological Science* **44**, 99–103 (2014).
32. Montgomery, J., Evans, J. A. & Wildman, G. 87Sr/86Sr isotope composition of bottled British mineral waters for environmental and forensic purposes. *Applied Geochemistry* **21**, 1626–1634 (2006).
33. Snoeck, C. *et al.* Towards a biologically available strontium isotope baseline for Ireland. *Science of The Total Environment* **712**, 136248 (2020).
34. Britton, K. *et al.* Sampling Plants and Malacofauna in 87Sr/86Sr Bioavailability Studies: Implications for Isoscape Mapping and Reconstructing of Past Mobility Patterns. *Front. Ecol. Evol.* **8**, (2020).
35. Wang, X. & Tang, Z. The first large-scale bioavailable Sr isotope map of China and

its implication for provenance studies. *Earth-Science Reviews* **210**, 103353 (2020).

36. Bataille, C. P., Crowley, B. E., Wooller, M. J. & Bowen, G. J. Advances in global bioavailable strontium isoscapes. *Palaeogeography, Palaeoclimatology, Palaeoecology* **555**, 109849 (2020).
37. Heaton, T. H. E. The 15N/14N ratios of plants in South Africa and Namibia: relationship to climate and coastal/saline environments. *Oecologia* **74**, 236–246 (1987).
38. Jackson, A. L., Inger, R., Parnell, A. C. & Bearhop, S. Comparing isotopic niche widths among and within communities: SIBER – Stable Isotope Bayesian Ellipses in R. *Journal of Animal Ecology* **80**, 595–602 (2011).
39. Chen, S. *et al.* Dietary evidence of incipient social stratification at the Dawenkou type site, China. *Quaternary International* **521**, 44–53 (2019).
40. Syväranta, J., Lensu, A., Marjomäki, T. J., Oksanen, S. & Jones, R. I. An Empirical Evaluation of the Utility of Convex Hull and Standard Ellipse Areas for Assessing Population Niche Widths from Stable Isotope Data. *PLOS ONE* **8**, e56094 (2013).
41. Dong, Y., Lin, L., Zhu, X., Luan, F. & Underhill, A. P. Mortuary ritual and social identities during the late Dawenkou period in China. *Antiquity* **93**, 378–392 (2019).
42. Hodson, F. R. Quantifying Hallstatt: Some Initial Results. *American Antiquity* **42**, 394–412 (1977).
43. Jørgensen, L. Family Burial Practices and Inheritance Systems : The Development of an Iron Age Society from 500 BC to AD 1 000 on Bornholm, Denmark. *Acta Archaeologica* (1987).
44. Flad, R. Ritual or structure? Analysis of burial elaboration at Dadianzi, Inner Mongolia. *Journal of East Asian Archaeology* **3**, 23–52 (2001).
45. Underhill, P. A. *An Analysis of Mortuary Ritual at the Dawenkou Site, Shandong, China*. (Journal of East Asian Archaeology, 2000).
