# Supplementary material for: Ancient DNA reveals a two-clanned matrilineal community in Neolithic China
Source: Nature. 2025 Jun 4;643(8074):1304–11. doi: 10.1038/s41586-025-09103-x (PMC12310535; doi:10.1038/s41586-025-09103-x)
Supplement: Supplementary file 2 — Reporting Summary [file 41586_2025_9103_MOESM2_ESM.pdf]

nature portfolio

Corresponding author(s): Chao Ning
Last updated by author(s): Apr 24, 2025

Reporting Summary

Nature Portfolio wishes to improve the reproducibility of the work that we publish. This form provides structure for consistency and transparency in reporting. For further information on Nature Portfolio policies, see our Editorial Policies and the Editorial Policy Checklist.

Statistics

For all statistical analyses, confirm that the following items are present in the figure legend, table legend, main text, or Methods section.

n/a	Confirmed	
☐	☒	The exact sample size (*n*) for each experimental group/condition, given as a discrete number and unit of measurement
☒	☐	A statement on whether measurements were taken from distinct samples or whether the same sample was measured repeatedly
☐	☒	The statistical test(s) used AND whether they are one- or two-sided<br>*Only common tests should be described solely by name; describe more complex techniques in the Methods section.*
☒	☐	A description of all covariates tested
☒	☐	A description of any assumptions or corrections, such as tests of normality and adjustment for multiple comparisons
☐	☒	A full description of the statistical parameters including central tendency (e.g. means) or other basic estimates (e.g. regression coefficient) AND variation (e.g. standard deviation) or associated estimates of uncertainty (e.g. confidence intervals)
☐	☒	For null hypothesis testing, the test statistic (e.g. *F*, *t*, *r*) with confidence intervals, effect sizes, degrees of freedom and *P* value noted<br>*Give P values as exact values whenever suitable.*
☐	☒	For Bayesian analysis, information on the choice of priors and Markov chain Monte Carlo settings
☒	☐	For hierarchical and complex designs, identification of the appropriate level for tests and full reporting of outcomes
☐	☒	Estimates of effect sizes (e.g. Cohen's *d*, Pearson's *r*), indicating how they were calculated

Our web collection on statistics for biologists contains articles on many of the points above.

Software and code

Policy information about availability of computer code

Data collection	Illumina sequence data were processed using the following programs to obtain genotype data used in the analysis: AdapterRemoval v2.3.1, BWA v0.7.17, Samtools v1.9, DeDup v0.12.2, bamUtils v1.0.13, pileupCaller (https://github.com/stschiff/sequenceTools), mapDamage v2.0.6, ANGSD v0.910, Schmutzi v1.5.1. These programs are publicly available.
Data analysis	Population genetic data analysis in this study was performed using the following publicly available programs: Haplogrep3, smartpca v18140, qp3Pop v435, qpDstat v755, READ v1.0, KIN v3.1.3(https://github.com/DivyaratanPopli/Kinship_Inference), ancIBD v.0.3a1(https://github.com/hringbauer/ancIBD), hapROH v0.64(https://haproh.readthedocs.io/en/latest/intro.html), GLIMPSE2.Non-default parameters used in our analysis are described in the Methods section. The base map in Figure 1 was generated by ArcGIS v9.2. Calibration of AMS 14C dating results was done by either IntCal20 or OxCal v4.4.0.

For manuscripts utilizing custom algorithms or software that are central to the research but not yet described in published literature, software must be made available to editors and reviewers. We strongly encourage code deposition in a community repository (e.g. GitHub). See the Nature Portfolio guidelines for submitting code & software for further information.

Data

Policy information about availability of data

All manuscripts must include a data availability statement. This statement should provide the following information, where applicable:

- Accession codes, unique identifiers, or web links for publicly available datasets
- A description of any restrictions on data availability
- For clinical datasets or third party data, please ensure that the statement adheres to our policy

Raw and alignment files (BAM format) are available at the Genome Sequence Archive in the National Genomics Data Center (GSA) under accession HRA007862(https://ngdc.cncb.ac.cn/gsa-human/browse/HRA007862). The previously reported ancient DNA datasets used in this study are available in Allen Ancient DNA Resource v.54.1 (https://reich.hms.harvard.edu/allen-ancient-dna-resource-aadr-downloadable-genotypes-presentday-and-ancient-dna-data). The dataset used for 14C radiocarbon date calibration are IntCal20 (http://intcal.org). The reference panel used for the Y haplogroup assignment is based on a current Y chromosome phylogenetic tree (http://yoogene.com/sourse/).. The Genome Reference Consortium Human Build 37 (GRCh37) is available through the National Center for Biotechnology Information under accession number PRJNA31257. The revised Cambridge reference sequence (rCRS) is available through the National Center for Biotechnology Information under NCBI Reference Sequence NC_012920.1.

Research involving human participants, their data, or biological material

Policy information about studies with human participants or human data. See also policy information about sex, gender (identity/presentation), and sexual orientation and race, ethnicity and racism.

Reporting on sex and gender	The genetic sex of our ancient samples was determined by analyzing the ratio of X and Y chromosome coverages relative to autosomes.
Reporting on race, ethnicity, or other socially relevant groupings	The concept of race or ethnicity was not applicable to our study, as we focused on ancient DNA samples where such social constructs do not apply. Instead, we classified our samples based on archaeological context and cultural associations with the Dawenkou culture. The categorization was done based on the site location, burial practices, and associated artifacts typical of the Dawenkou culture, as identified through archaeological methods.
Population characteristics	Our study examined individuals from the Neolithic period, specifically those associated with the Dawenkou culture in China. The samples represent a population that lived approximately 4000-5000 years ago, based on radiocarbon dating of the burial context. The demographic characteristics such as age at death were inferred from osteological analysis. Detailed genetic information was obtained through sequencing ancient DNA, revealing insights into the genetic structure and diversity of this ancient population.
Recruitment	Participants were not recruited in the traditional sense, as this study involved ancient human remains. The samples were obtained from well-documented archaeological excavations conducted by the Shandong Institute of Cultural Relics and Archaeology. We ensured that all samples were ethically sourced and that the excavation and analysis followed proper archaeological and bioethical guidelines to minimize contamination and ensure respectful handling of human remains.
Ethics oversight	The study protocol was approved by the ethics committee of the Shandong of Cultural Relics and Archaeology. Additionally, all necessary permits for the excavation and analysis of the ancient human remains were obtained from relevant local authorities.

Note that full information on the approval of the study protocol must also be provided in the manuscript.

Field-specific reporting

Please select the one below that is the best fit for your research. If you are not sure, read the appropriate sections before making your selection.

☐ Life sciences ☐ Behavioural & social sciences ☒ Ecological, evolutionary & environmental sciences

For a reference copy of the document with all sections, see nature.com/documents/nr-reporting-summary-flat.pdf

Ecological, evolutionary & environmental sciences study design

All studies must disclose on these points even when the disclosure is negative.

Study description	This study includes whole genome sequencing of 60 ancient individuals from Shandong province, northeast China, dating between 2,750 and 2,500 BCE. 46 individuals were from the southern cemetery of the Fujia archaeological site and the rest 14 were from the northern cemetery.
Research sample	Research samples are composed of 60 ancient genomes from the Fujia archaeological site in northeastern China. The samples are dated between 2,750 and 2,500 BCE, associated with the Dawenkou culture.
Sampling strategy	No sample-size selection was performed prior to the study. To produce ancient genomes reported in this study, we screened the accessible skeletal elements from the relevant geographic regions and time periods, and produced in-depth sequencing data for those with sufficient endogenous DNA preservation and without substantial contamination.

Data collection	Sequencing of the libraries was performed on an Illumina NovaSeq instrument at the Novogene Company, China, in the 150-bp paired-end sequencing design.
Timing and spatial scale	Laboratory works and sequencing was conducted over the period from February 2022 to December 2023. Samples were taken from northest China, the locations of which are provided in Fig. 1.
Data exclusions	We excluded samples only if the samples do not meet the quality criteria, either by having low level of endogenous human DNA prohibiting genome-scale sequencing or by showing high level of contamination estimates. For population genetic analysis that requires exclusion of genetic relatives, we excluded closely related individuals (1st degree relatives) by removing one with lower coverage from each pair.
Reproducibility	We took multiple individuals from each archaeological site, if available, to support the representativeness of their genetic profiles. For each sample, we estimated contamination level to support the authenticity of data.
Randomization	Ancient genomes were first analyzed by each individual, and then were allocated into the analysis group based on their archaeological context, absolute date (14C dating), and their individual genetic profile. Randomization is not applicable.
Blinding	There was no experimental treatment of samples involved in this study that requires blinding. Data analysis was performed based on the analysis groups that were defined by external information (archaeological context and date).

Did the study involve field work? ☐ Yes ☒ No

Reporting for specific materials, systems and methods

We require information from authors about some types of materials, experimental systems and methods used in many studies. Here, indicate whether each material, system or method listed is relevant to your study. If you are not sure if a list item applies to your research, read the appropriate section before selecting a response.

Materials & experimental systems

n/a	Involved in the study	
☒	☐	Antibodies
☒	☐	Eukaryotic cell lines
☐	☒	Palaeontology and archaeology
☒	☐	Animals and other organisms
☒	☐	Clinical data
☒	☐	Dual use research of concern
☒	☐	Plants

Methods

n/a	Involved in the study	
☒	☐	ChIP-seq
☒	☐	Flow cytometry
☒	☐	MRI-based neuroimaging

Palaeontology and Archaeology

Specimen provenance	The specimens analyzed in this study were obtained from the Fujia archaeological site, which is associated with the Dawenkou culture in Shandong province, China. Permits for the excavation and export of these samples were obtained from the Shandong Institute of Cultural Relics and Archaeology.
Specimen deposition	The specimens analyzed in this study have been deposited at the ancient DNA laboratory at Peking University. These specimens are available for further research upon request, under the guidelines and supervision of the Shandong Institute of Cultural Relics and Archaeology, to permit free access by other researchers.
Dating methods	New radiocarbon dates were provided for this study. The samples were pretreated, and measurements were taken at the Radiocarbon Dating Laboratory of Peking University and Beta, Inc.. The calibration program used were OxCal v4.4 and IntCal20, and quality assurance protocols were strictly followed as per the laboratory standards. No new dates are provided beyond those obtained during this pretreatment and measurement process.

☒ Tick this box to confirm that the raw and calibrated dates are available in the paper or in Supplementary Information.

Ethics oversight	The study protocol was approved by the ethics committee of the Shandong Institute of Cultural Relics and Archaeology. Additionally, all necessary permits for the excavation and analysis of the ancient human remains were obtained from relevant local authorities.

Note that full information on the approval of the study protocol must also be provided in the manuscript.

Plants

Seed stocks	We collected wild plant specimens from within a 50 km radius of the Fujia archaeological site. The specific locations, dates, and sampling procedures were meticulously recorded to maintain data integrity and reproducibility.
Novel plant genotypes	N/A (No novel plant genotypes were produced or used in this study).
Authentication	We conducted strontium isotope analysis (87Sr/86Sr) on the collected wild plant specimens to authenticate their geographical origin and ensure they were representative of the local environment. These isotopic measurements were compared with known values from the region to confirm their accuracy.
